# Analysis of the autopsy, toxicological, and psychiatric reports of Portugal’s first major forensic case: part III

**DOI:** 10.1080/20961790.2021.1898079

**Published:** 2021-05-07

**Authors:** Ricardo Jorge Dinis-Oliveira

**Affiliations:** a Department of Public Health and Forensic Sciences, and Medical Education, Faculty of Medicine, University of Porto, Porto, Portugal; b IINFACTS—Institute of Research and Advanced Training in Health Sciences and Technologies, Department of Sciences, University Institute of Health Sciences (IUCS), Advanced Polytechnic and University Cooperative (CESPU), CRL, Gandra, Portugal; c UCIBIO-REQUIMTE, Laboratory of Toxicology, Department of Biological Sciences, Faculty of Pharmacy, University of Porto, Porto, Portugal

**Keywords:** Forensic sciences, forensic reports, alkaloids, history of legal medicine, Flores Street, Vicente Urbino de Freitas, José António de Sampaio Junior, Mário Guilherme Augusto de Sampaio

## Abstract

This work presents an odd historical record obtained through more than 14 years of research regarding one of the first major European forensic cases. The presumed homicide of Mário Guilherme Augusto de Sampaio in 1890 was allegedly perpetrated by his uncle, the prestigious doctor Vicente Urbino de Freitas. This famous poisoning had international repercussions for decades, with the participation of several forensic experts that made the history of forensic sciences, namely forensic toxicology and pathology. This third work aims to collect, restore, and analyse all the forensic evidence, particularly from the autopsy, toxicological, and psychiatric forensic reports. Facts regarding the life of Vicente Urbino de Freitas during his exile in Brazil were also recovered, along with a vast and outstanding assortment of forensic medicine photographs from the 19th century.

## Introduction

**Figure 1.**
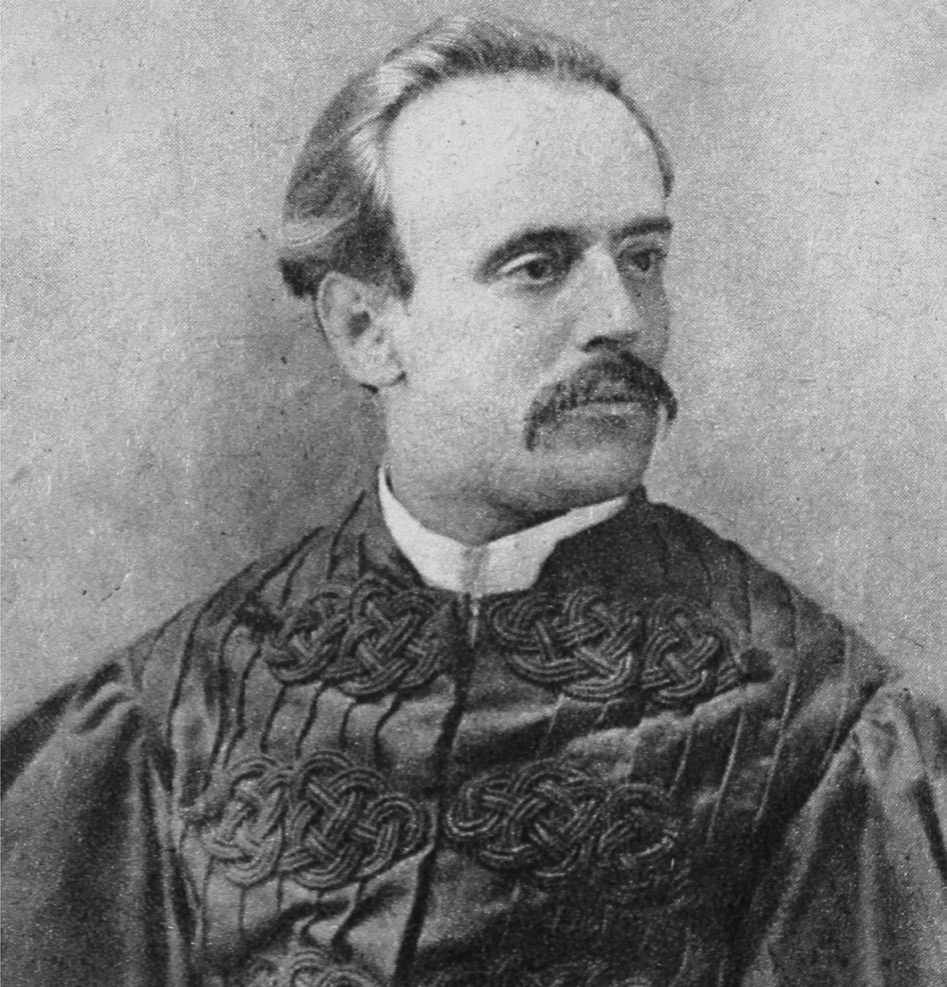
Recovered portrait of Vicente Urbino de Freitas in Brazil during the golden times.

**Figure 2.**
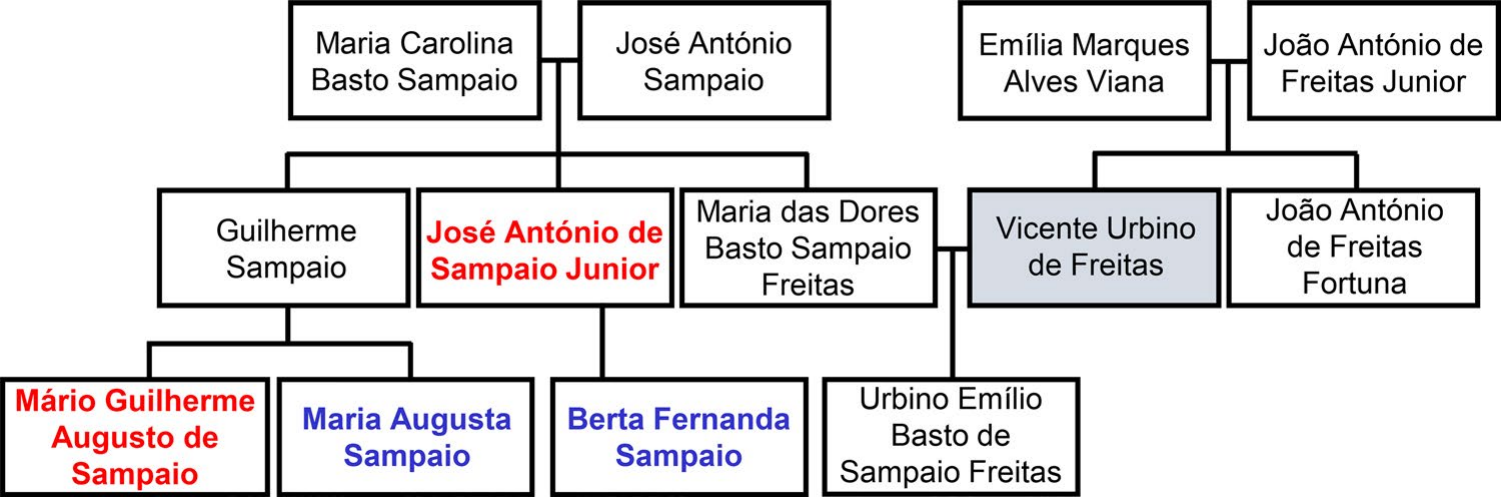
The genealogy of some of the main characters of the Vicente Urbino de Freitas forensic case. Fatal and non-fatal alleged intoxicated victims are written in red and blue, respectively.

In the vast gallery of infamous criminals that have been brought to justice, the poisoner Vicente Urbino de Freitas (Figure 1) is one of the most well-known [1–3]. He was a noted physician with regular scientific productivity [4,5]. However, nothing made him more famous than when he was convicted of the fatal poisoning of his nephew, Mário Guilherme Augusto de Sampaio, who died on 2 April 1890. This crime had incomparable international repercussions, even rivalling large judicial cases. The first major medicolegal case in Portugal both fascinated and stunned Portuguese society in the late 19th century [1,2]. The genealogy of the most relevant personalities in this forensic case is shown in Figure 2.

One of the most contested procedural elements of the defence was the discrepancy between expert opinions on forensic reports, in particular the toxi­cological examinations. The same data led to diametrically opposed conclusions. Specialists from Coimbra and abroad, mostly Germans, reacted nega­tively to Porto experts’ conclusions, even writing that the “forensic report of the Porto experts was a shame. There are only errors and impurities in it. Due to ignorance or slouch, they miserably cheated justice. The analysis did not show the presence of any toxic substance…”

Judgments among physicians regarding the toxic substances that caused the poisoning also diverged. Indeed, the symptoms evidenced by the three children (i.e. Mário Guilherme Augusto de Sampaio, Berta Fernanda Sampaio and Maria Augusta Sampaio) and partly by their grandmother Maria Carolina Basto Sampaio, such as asthenia, vertigo, nausea, vomiting, deafness, drowsiness, and urinary retention, are common to many poisonings. Therefore, only a qualitative and quantitative toxicological analysis of the biological samples taken during the autopsy could clarify the cause of death. Some doctors suspected morphine, eserine (i.e. physostigmine), or both. It is worth remembering that after they ingested the almonds and chocolate cakes on 31 March and were given Eno® salts, the children vomited and were relieved. They woke up perfectly restored on 1 April. After the symptoms’ remission, it was very strange to see a worsening of their clinical status on the morning of 2 April. Therefore, the suspicion was grounded on an alleged repetition of the criminal administration of the poison(s) incorporated into the cakes and/or Easter almonds, or the subsequent administration of other xenobiotics. Then, the physicians collectively asked the family if they took other medications. In her good faith and without any suspicion, Maria Carolina Basto Sampaio did not disclose the apple cider vine­gar enemas prepared and applied by the hands of Vicente Urbino de Freitas to the children in the evening and in the early morning. Only this good faith could explain why she gave negative answers to the questions of doctors. Shortly thereafter, Maria Carolina Basto Sampaio and her maid, Maria Luiza, disclosed the facts. Since then, it became plausible that Vicente Urbino de Freitas purposely hid these and other details from the clinicians. Was that on purpose? They probably thought so.

This third work on the first major Portuguese medi­colegal case aims to collect, restore, and analyse all the autopsy, toxicological, and psychiatric reports. Facts regarding the life of Vicente Urbino de Freitas during his exile in Brazil were also recovered. This work presents an odd historical forensic record obtained through more than 14 years of research using books, newspapers, scientific journals, interviews of different individuals, and forensic reports scattered around the world [1,6–14]. Of note, this manuscript also compiles a vast and outstanding assortment of photographs. For a better understanding of the forensic case described in this work, the previous publications of the author should be considered [1,2].

## Materials and methods

The bibliographical research inherent to this reconstruction began in the middle of 2007 and was performed as previously described [1,2]. Several sources were used to discover historical documents of the case, such as the Portuguese Centre of Photography, Moreno and Parente Pharmacies of Porto, Azores North House, Grande Hotel de Paris, Edições Afrontamento®, *Jornal do Brasil*, Libraries of the Faculties of Arts, Sciences, Law and Medicine of the University of Porto, Portuguese Association of Photography, Angra do Heroísmo Museum, Archive Division of the Santa Cruz do Bispo Prison, Camilo House-Museum, Court of Appeal of Porto, Historical Archive of Abreu Travel Agency, National Library of Brazil, Library of Health Sciences Faculty of the University of Coimbra, Lisbon Municipal Library, Aurélia de Sousa School Library of Porto, Póvoa do Varzim Municipal Library, Agramonte Cemetery, Library of the University Centre of the Campanha Region – URCAMP, Museum of Natural History and Science of the University of Porto, Portuguese National Library, Porto Municipal Library, Rui Barbosa House Foundation of Rio de Janeiro, and Ordem do Carmo Cemetery, among other Portuguese, Brazilian, French, Spanish, Uruguayan, English, and German sources (given the worldwide coverage of this case). Interviews of the living relatives of the main characters involved in the case were also carried out.

## Autopsy reports of Mário Guilherme Augusto de Sampaio

**Figure 3.**
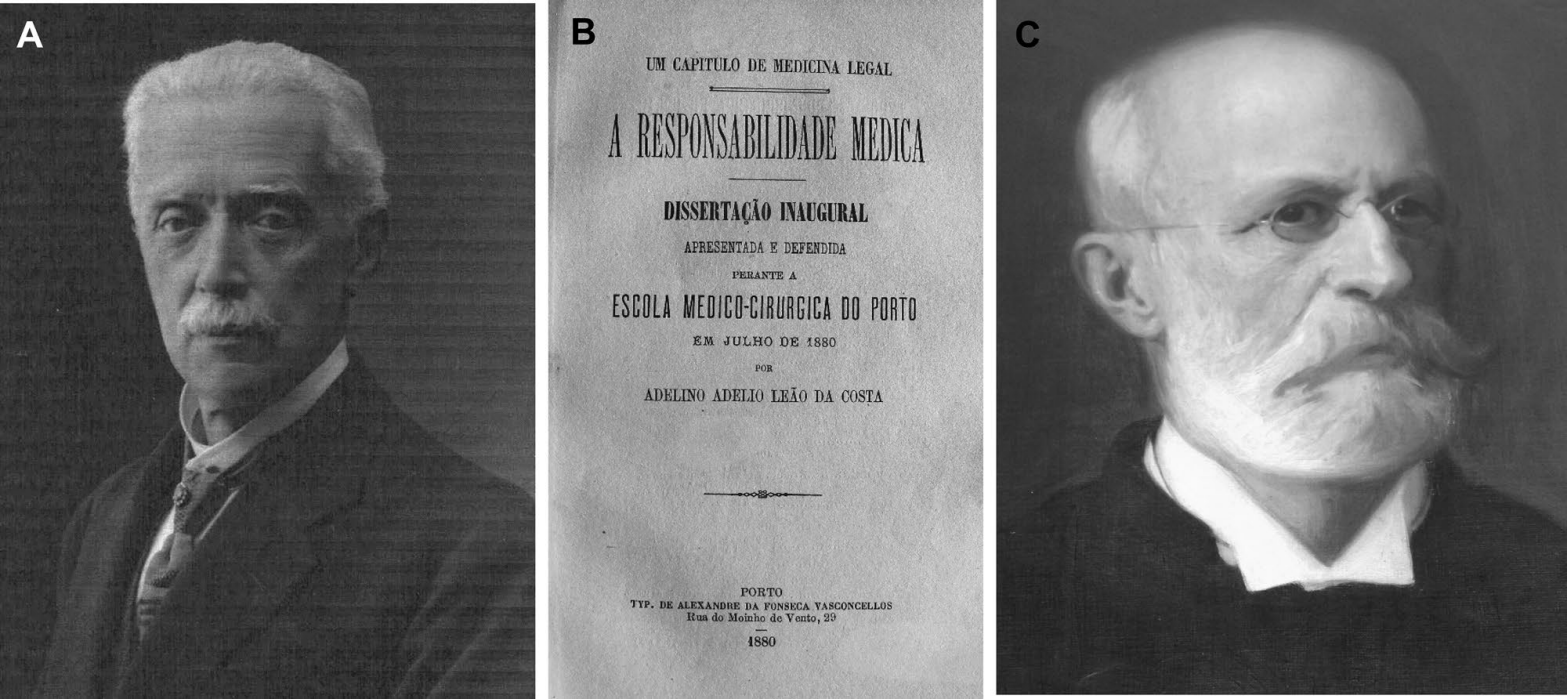
(A) The recovered portrait of Júlio Estevão Franchini. (B) The cover of the Adelino Adélio Leão da Costa dissertation. (C) The recovered portrait of Agostinho António do Souto.

On 4 April 1890, approximately 44 h after death, in the cemetery of Agramonte, and in the presence of Judge Bento José da Silva Lima, an autopsy was ordered to medical surgeons Júlio Estevão Franchini (1854–1932; Figure 3A) [15] and Adelino Adélio Leão da Costa (Figure 3B provides a copy of his dissertation). Júlio Estevão Franchini is considered by many to be one of the great physicians responsible for modern surgery in Portugal and was one of the doctors who confirmed the death of the daughter of Vicente Urbino de Freitas, who died on 16 November 1893. Adelino Adélio Leão da Costa also appeared in the Flores Street house after Mário Guilherme Augusto de Sampaio’s death and is considered the first specialist in genitourinary diseases in Porto. Mário Guilherme Augusto de Sampaio, 14 years old and son of Guilherme Augusto Basto de Sampaio and Rosa Olympia de Brito Sampaio, already deceased, was lying on the anatomi­cal table, dressed in a jacket, pants, black vest, long johns, white shirt with tie, black socks, and polished shoes. Forensic results are detailed in Table 1.

Already in a lead coffin, a second autopsy was performed on 17 April by the two doctors responsible for the first autopsy, together with Doctor Agostinho António do Souto (1825–1911; Figure 3C), professor of organic chemistry at the Medical-Surgical School of Porto. He was also responsible for the toxicological forensic analysis (see ahead). In the second forensic autopsy, heart, lungs, remaining intestines, kidneys, and blood were collected and in this autopsy the samples were not stored in ethanol.

**Table 1.** Results of the first forensic autopsy of Mário Guilherme Augusto de Sampaio.

Forensic autopsy	Results
External habit	
	• Livor mortis in the dorsal region of the trunk and upper and lower limbs • Reddish spots on the dorsal region, compatible with rubefacient agents applied antemortem • Bloody foam coming out through the nose and mouth
Internal habit	
Cranial cavity	• Extensive and generalised meningeal, arterial, and venous congestion extending to the inner surface of skull • Numerous miliary haemorrhagic foci appearing along the vessels • The opening of the upper longitudinal sinus resulted in an outflow of a large quantity of partly coagulated black blood • The section of the brain exhibited the same intense vascular congestion, extending to the medulla oblongata • Examination of the spine was not performed due to the absence of adequate instrumentation
Thoracic cavity	• Absence of abnormalities in the anterior mediastinum • Normal pericardial fluid without bleeding, abnormal pigmentation, bruising or milky plaques • Perfectly normal external surface of the heart correlating to the age of the victim • Opening of the cardiac cavities in situ showed the following: i) Right ventricle with black fluid blood, normal internal surface and preserved integrity of the tricuspid valve; ii) Right auricle filled with black and partly coagulated blood. The large clot on the auriculoventricular orifice was soft, elastic, and reddish yellow in colour. Nothing abnormal on the walls of the auricle or in the holes of the vena cava; iii) Left ventricle was filled with black fluid blood and stopped in diastole. Perfect integrity of the mitral and aortic valves was noted. This perfect integrity of the valves was later confirmed by the water test. The inner surface of the ventricle and auricle were perfectly normal • After excluding the absence of bilateral pleural effusion, the heart, the lungs and trachea, were removed *en bloc* • The lungs were perfectly permeable and normal, but heavily congested • In the trachea, larynx and thick bronchi, abundant bloody foam was noted
Abdominal cavity	• Absence of abnormal adhesions between the different organs • Gastric and duodenal contents, urine, brain, cerebellum, stomach, intestine, liver, and pancreas were collected for toxicological and histological examination. The viscera were preserved in ethanol • Bladder contained approximately 170 mL of urine • The inner surface of the stomach was covered by a viscous liquid of yellow colour, slightly greenish and adherent • On the surface of the longitudinal folds of the stomach and duodenum, a vascular congestion was perfectly clear, similar to that of the brain meninges • Slate-coloured liver both on its surface and in the different cuts • In the spleen and kidneys, nothing was noticed that was worthy of special mention

## Autopsy reports of José António de Sampaio Junior

**Figure 4.**
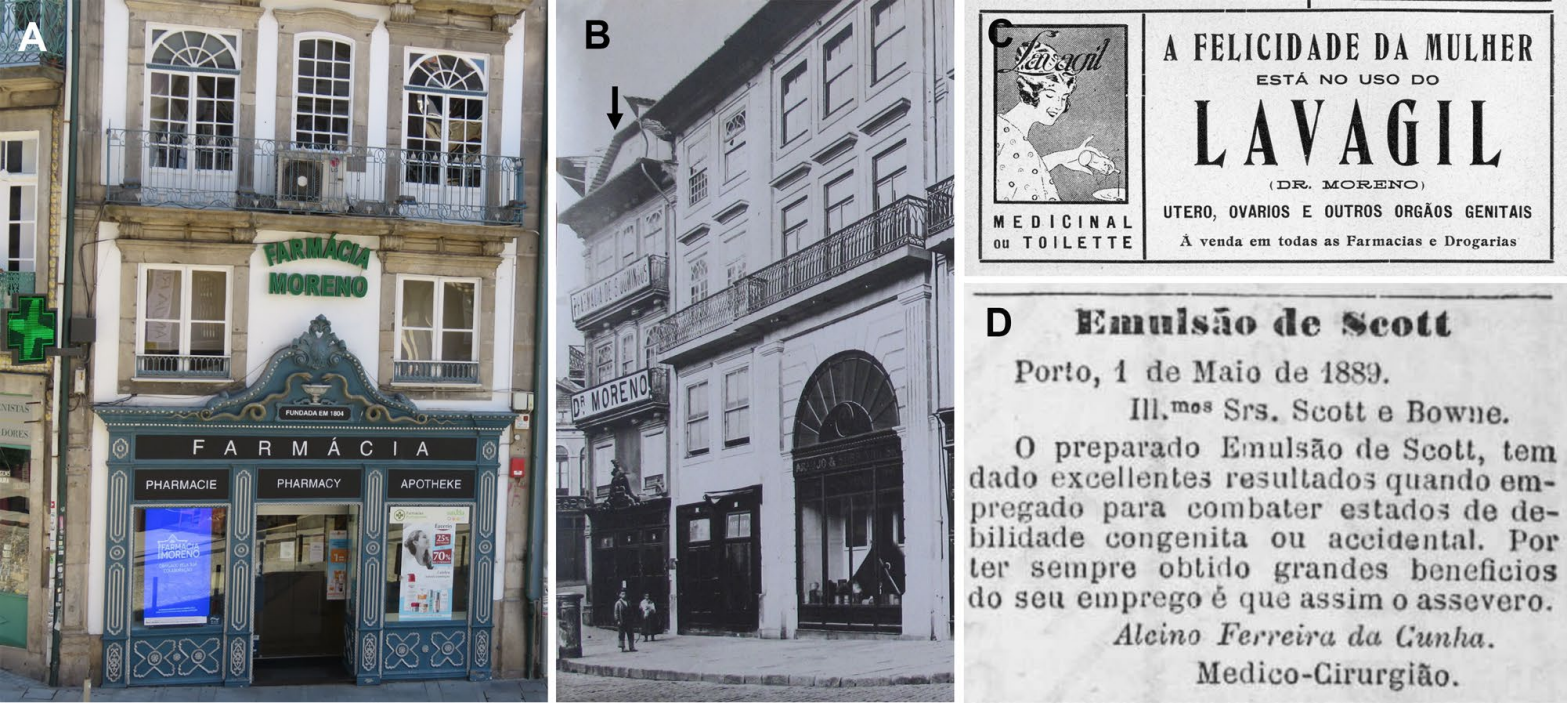
Moreno Pharmacy at Porto today (A) and in 1950 (B) (see arrow). Rodrigo de Sousa Moreno was a surgeon and pharmacist and the first director of the Moreno Pharmacy. This pharmacy is the second oldest in the city. Founded in 1804 with the previous name of *Drogaria Félix & Filho* (owned by Félix da Fonseca Moura, Professor of Pharmacy and Toxicology of the Medical-Surgical School of Porto), it was dedicated to the production of products of plant and animal origin to treat diseases. In 1890, it was acquired by Rodrigo de Sousa Moreno, and the name was changed to Pharmacy S. Domingos. The name Moreno Pharmacy, as it is currently known, dates to 1928 when the pharmacy passed to his nephew António Moreno. Exclusive pharmaceutical formulas were produced in the laboratories on the upper floors and a very well-preserved pharmacy museum (https://www.farmaciamoreno.pt/pt/museu) can be seen today. Some pictures of medicines advertised by (C) Rodrigo de Sousa Moreno and (D) Alcino Ferreira da Cunha as warrants of quality.

On 10 April 1890, in the cemetery of Agramonte and in the presence of both Judge Bento José da Silva Lima and the Public Prosecutor Miguel Maria Guimarães Pestana da Silva, the exhumation was ordered and the autopsy was performed by Doctors Rodrigo de Sousa Moreno (1851–1920; Figure 4A,B) and Alcino Ferreira da Cunha (Figure 4C). José António de Sampaio Junior had been buried since 4 January 1890 in the Order of Our Lady of Carmo in the Family Deposit “Almeidas”, n°20. The alleged victim was the widower of Cacilda d’Almeida, who died in 1874, daughter of Constantino d’Almeida and Cândida Marques dos Santos Carregal d’Almeida, also dead at the time. These data were particularly important for finding the victim’s remains in Porto, and this fact will be explored in future studies. He was inside two coffins, the exterior of lead and the interior of pine planks, and both were then opened. The victim was dressed and in a supine position with the legs and abdomen encased in a thick layer of plaster and the face, neck, and hands appearing violet in colour and swollen. Having been buried for three months and eight days, they found him “in an advanced state of putrefaction”, making it impossible to observe any signs of injury relating to a cause of death [12,13]. The toxicology analysis revealed inconclusive results, a fact attributed to the putrefactive alterations. After the autopsy, José António de Sampaio Junior was re-enclosed in a lead box and returned to the same grave. Forensic autopsy results are detailed in Table 2.

**Table 2.** Forensic autopsy results of José António de Sampaio Junior.

Forensic autopsy	Results
External habit	• Seemingly robust structure, with hair and moustache, and no beard • Evidence of dilated abdominal cavity compatible with the production of utrefaction gases • Violaceus colour with epidermis detached at some points • No signs of trauma, although difficult to characterise due to signs of putrefaction
Internal habit	• Semiliquid brain mass • Impossibility of forensic evaluation of the thoracic and abdominal cavities due to the advanced state of decomposition, with the spleen and part of the intestine absent • Brain mass, stomach, rest of the intestine, liver, kidneys, lungs, and heart were harvested for toxicological analysis for morphine and other alkaloids

## Official toxicological experts

**Figure 5.**
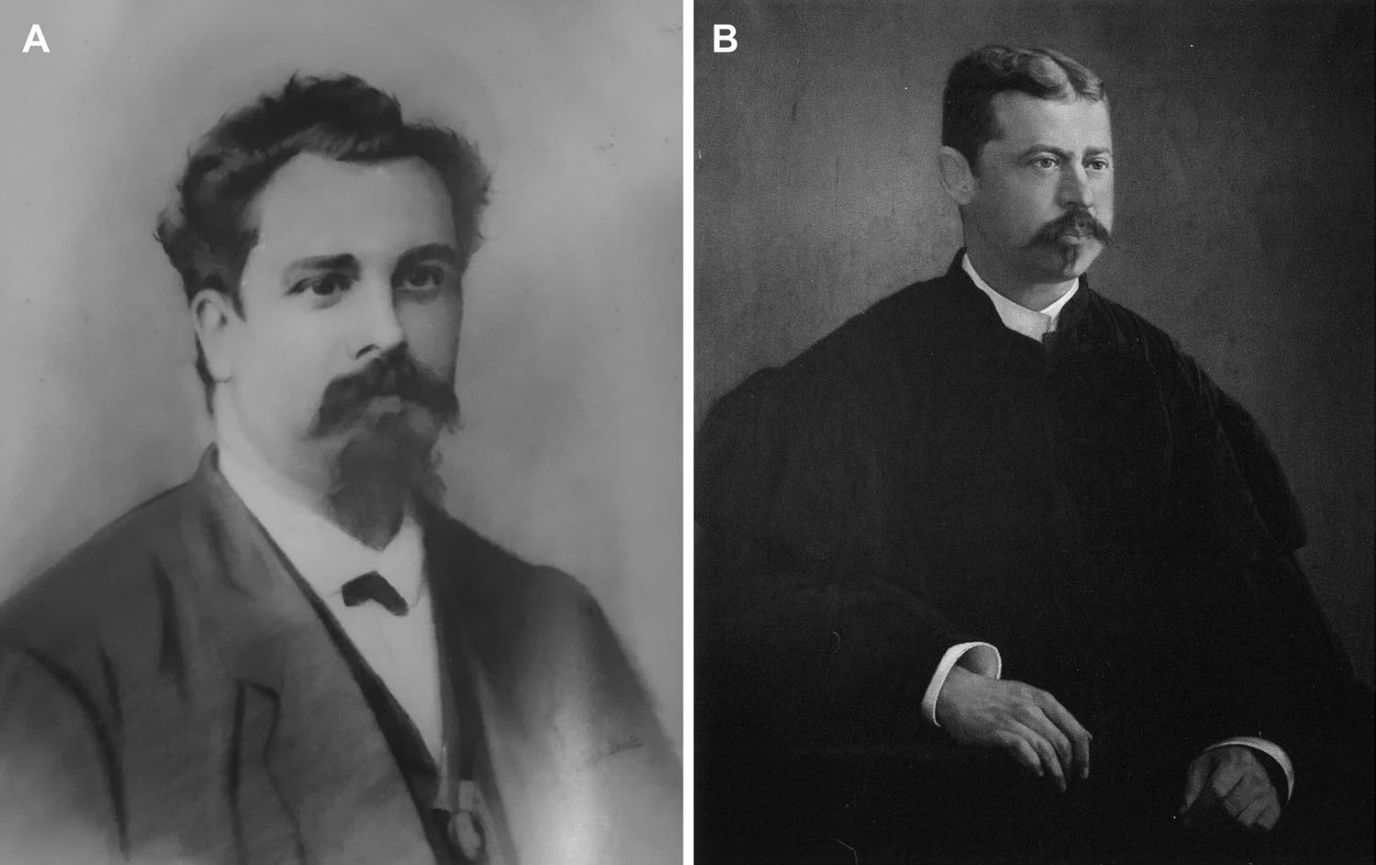
Recovered portraits of (A) Joaquim Pinto de Azevedo and (B) Manuel Rodrigues da Silva Pinto.

**Figure 6.**
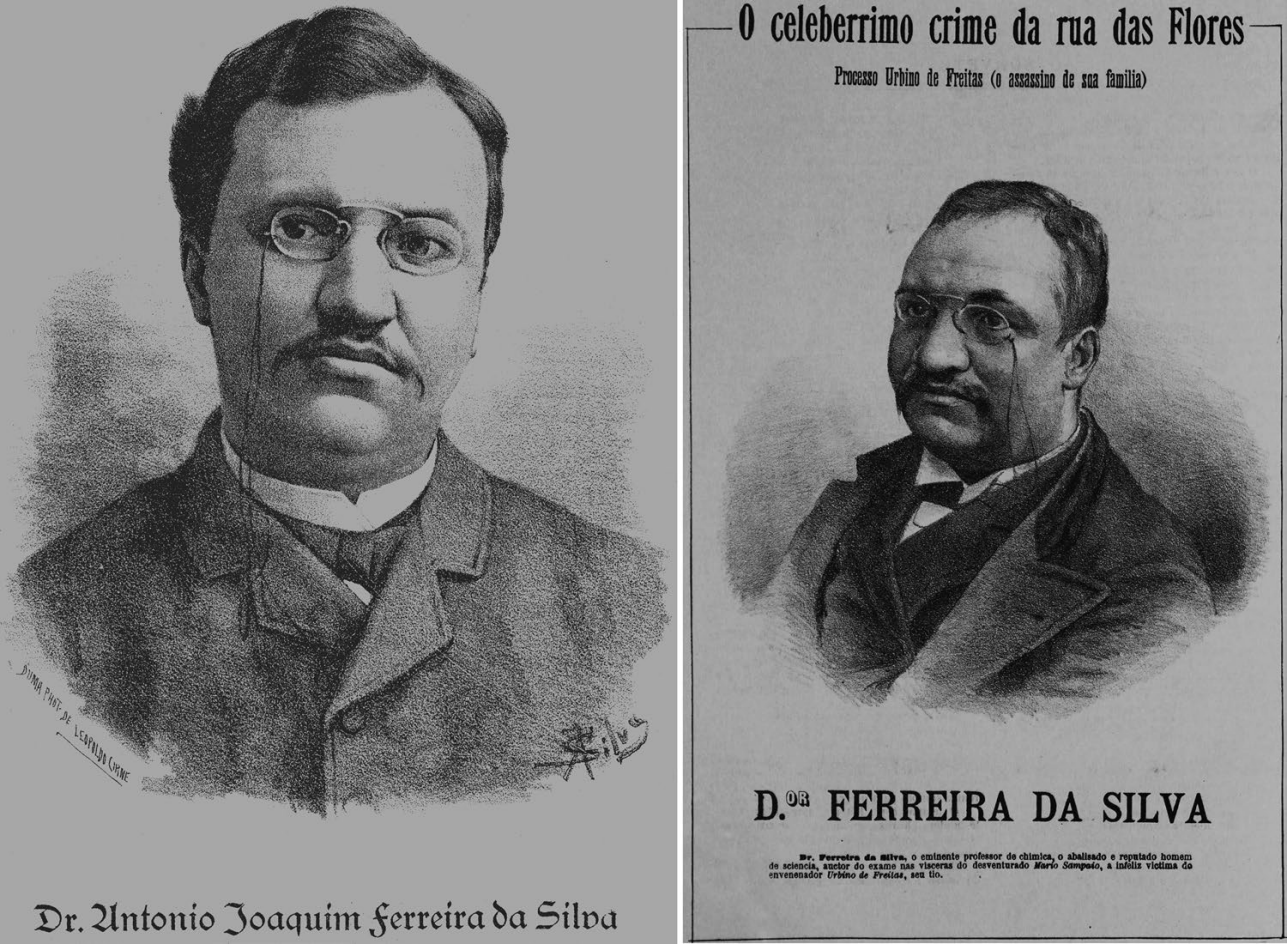
Recovered portraits of António Joaquim Ferreira da Silva.

**Figure 7.**
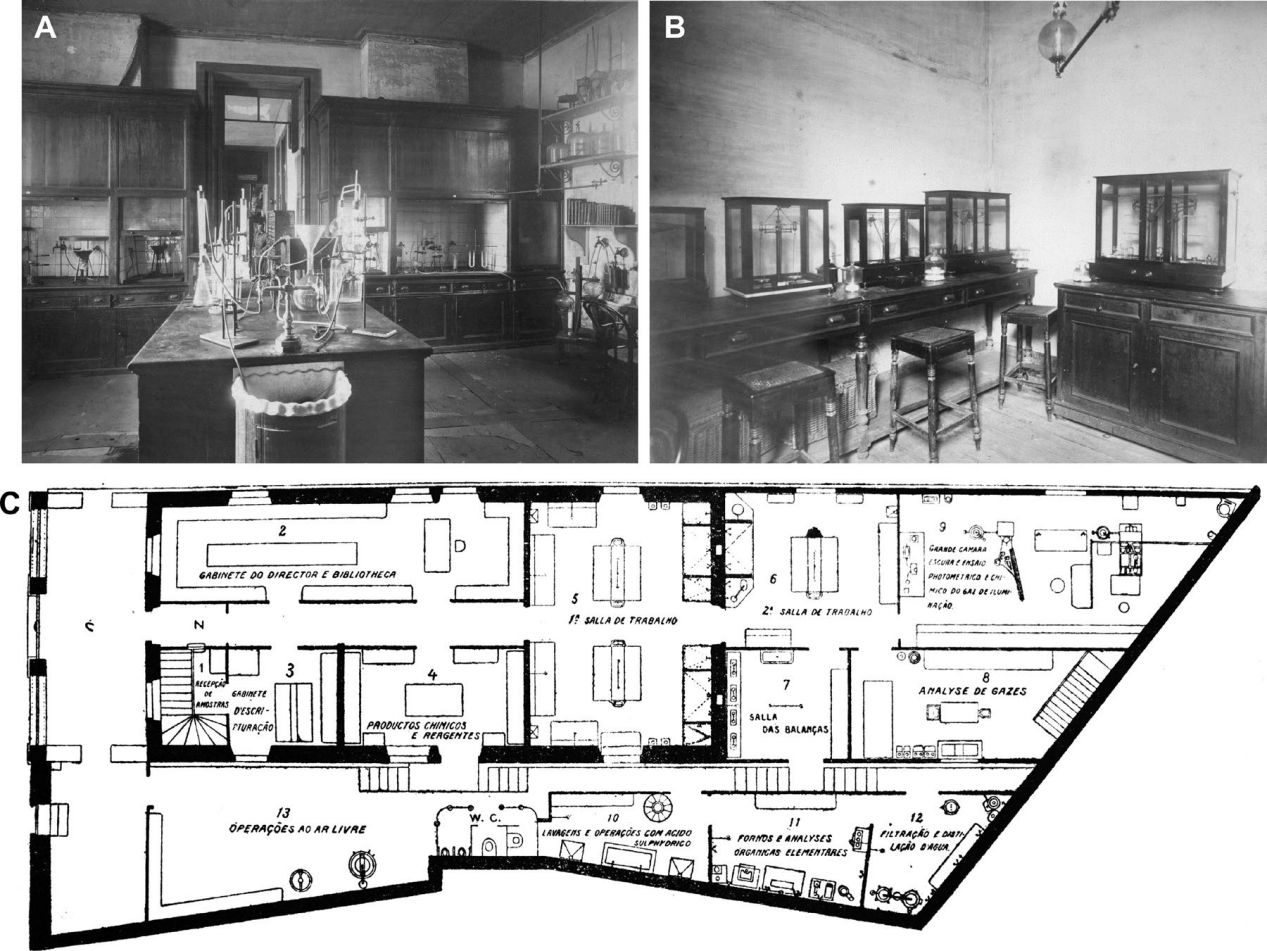
(A) Room n°5 of the Municipal Chemistry Laboratory of Porto containing two chimneys closed by glazed windows and (B) room n°7 containing laboratory scales. The architectural plan of the laboratory dating from 1916, is also provided (C).

**Figure 8.**
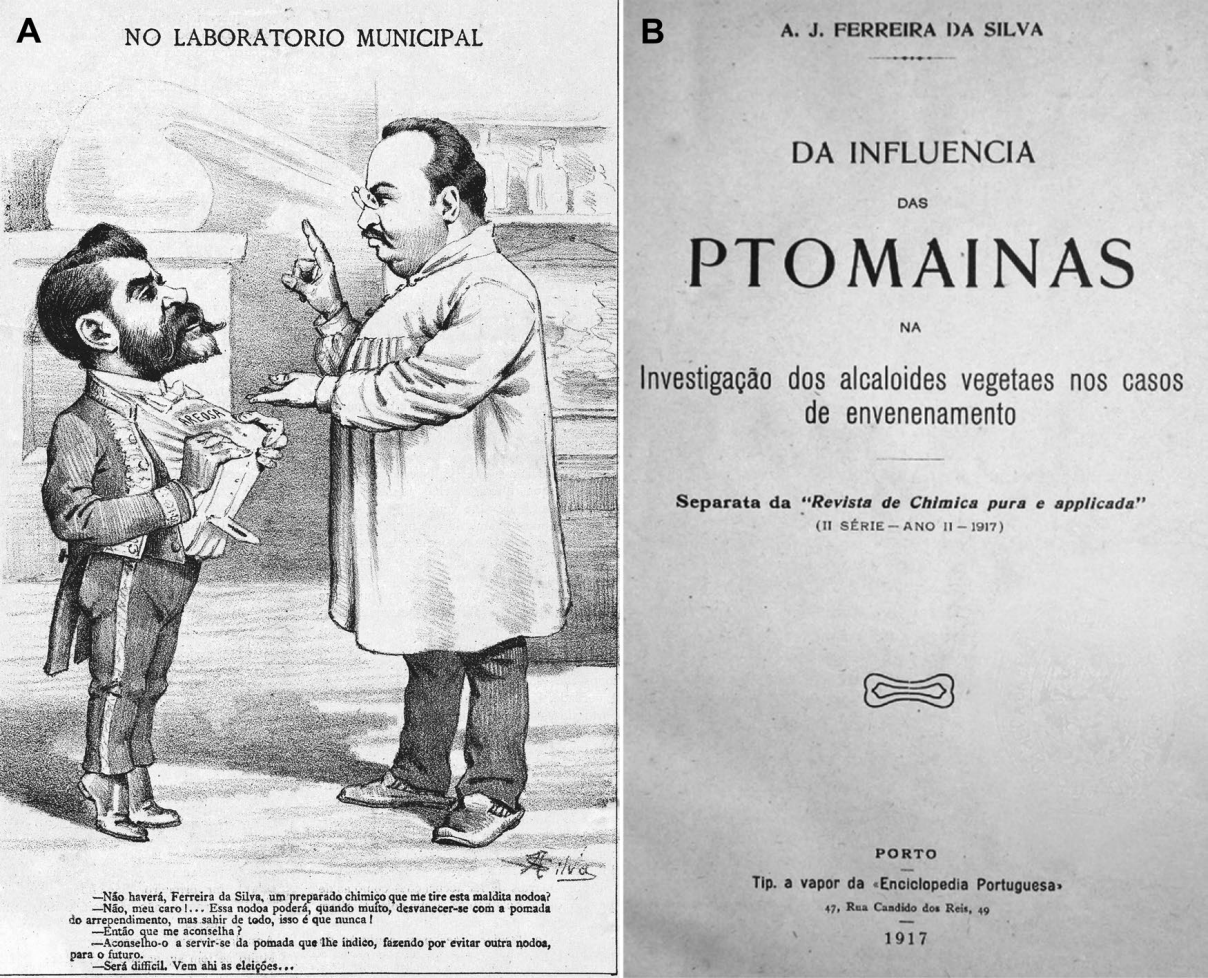
Recovered cartoon of António Joaquim Ferreira da Silva in routine chemistry work (A) and a copy of the cover of his work in ptomaines dating from 1917 (B).

At the heart of this case was the use of toxicologi­cal analyses in court. The main forensic issues involved toxicological analyses of the corpses and the suspected poisoned foods [7]. A group of four experts was assembled based on their virtue and honesty of character, a guarantor of the actions of good men: i) Agostinho António do Souto (Figure 3C); ii) Joaquim Pinto de Azevedo (1841–1898; Figure 5A), who was a surgeon and anatomy preparator at the Medical-Surgical School of Porto; iii) Manuel Rodrigues da Silva Pinto (1850–1895; Figure 5B), who was a professor of legal medicine, public and private hygiene, and toxicology at the Medical-Surgical School of Porto. Manuel Rodrigues da Silva Pinto was also a professor of Portuguese at the Lapa College of the illustrious Portuguese doctor Ricardo de Almeida Jorge, who later replaced him as the head of the area of public hygiene and legal medicine. Today, there is a “Professor Rodrigues Pinto Award” assigned to the student with the best average score in the disciplines of Epidemiology and Legal Medicine; and iv) António Joaquim Ferreira da Silva (1853–1923; Figure 6), who was a professor and the Director of the Polytechnic Academy of Porto (once located where now the Rectory of the University of Porto is located), director of the Municipal Chemistry Laboratory of Porto (Figure 7), and founder of the Portuguese Chemical Society in 1911. Of the four experts, António Joaquim Ferreira da Silva deserves special mention since he was the leader of the very important toxicological works. Indeed, although criticisms for all experts were made, he was the target of specific attacks because of his special knowledge and proven technical and scientific quali­fications. The recovered cartoon of António Joaquim Ferreira da Silva in routine chemistry work and a copy of the cover of his work in ptomaines (i.e. “the animal alkaloids”) dated from 1917 are presented in Figure 8.

### Forensic literature

In 1892, following the publication of a book by a group of experts from Coimbra (see below) that compiled all the arguments in favour of the defendant [16], Agostinho António do Souto published a rebuttal to those reports contained in the supplements to the *Coimbra Médica* journal [17]. The four official experts edited a more complete book one year later [18], which was attached to the judicial process at the request of the authors. The book quickly sold out in approximately two months (pre­face of 20 February 1893), and in that same year, a new edition comprising 542 pages and 10 figures expanded the previous edition [12]. A French version was also produced [13] and represents a scientific work that all interested in forensic sciences should keep in their libraries. In this book, the experts explained all the methods used in the ana­lysis of the viscera and other physical suspicious samples (i.e. almonds, stamps, syringe, mattress, enemas), extensively transcribed all reports presented in court, and repelled and refuted the criticisms of the Portuguese and foreign contradictors. Like the first, this much more complete second edition was structured in three parts, as described below.

**Figure 9.**
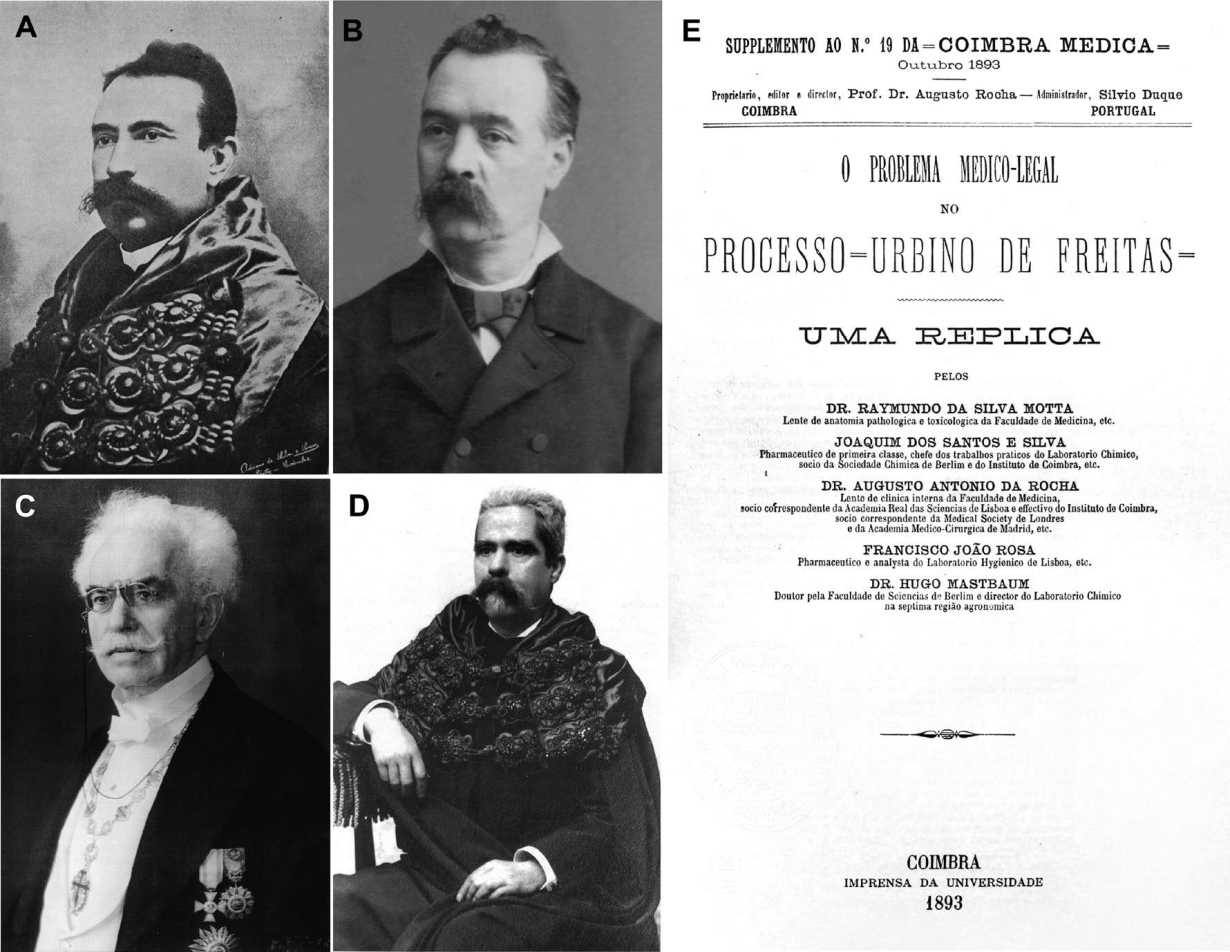
Recovered portraits of (A) Augusto António Rocha, (B) Joaquim dos Santos e Silva, (C) Hugo Mastbaum, and (D) Raimundo da Silva Mota. (E) The cover of the supplement to the *Coimbra Médica* n°19.

The first part, with 186 pages, is dedicated to comments and replies to the contradictors of the scientific *Coimbra Médica* journal (1881–1992) and the *Jornal de Pharmacia e Chimica*, primarily highlighting the unethical performance of Augusto António Rocha (1849–1901; Figure 9A), who was used as an “instrument of the defence”. He was a physician and professor of internal clinics from the Faculty of Medicine of the University of Coimbra and the principal editor of the *Coimbra Médica* journal (1881–1992). As declared by Augusto António Rocha himself, he accepted this difficult task because he had been a former faculty colleague of Vicente Urbino de Freitas for 8 years. The Porto experts condemned this conflict of interests and inspired by negative feelings about the truth and justice, rejected all “rough, frivolous and insulting criticisms” while underlining their total independence.

The second part, with 108 pages, includes all the toxicological of the suspicious biological samples belonging to Mário Guilherme Augusto de Sampaio, the liquids contained in the vials seized at Vicente Urbino de Freitas’ home, some almonds, the stamps, the syringe, José António de Sampaio Junior’s viscera, and the mattress in which José António de Sampaio Junior died. Moreover, it includes a series of 46 notes on products, criteria, reactions, and interpretations in the field of chemi­cal analysis.

Finally, the third part, with 240 pages, presents documents describing the circumstances of the case (e.g. petition of querela, pronunciation order, narrations); testimonials from physicians; autopsy results; theoretical texts on the signs, symptoms, and physiological effects of the various alkaloids under analysis; documents emanating opinions from experts (statements to the press, letters to national physicians and international experts); criticisms, texts from the *Coimbra Médica* journal and other publications; analytical notes applied to alkaloid investigation by António Joaquim Ferreira da Silva and international authors; texts of the new medical conference of 1892; judicial documents relating to medical-legal examinations; and the private letters of António Joaquim Ferreira da Silva that substantiated the famous “inciden te Dragendorff” (translated to “the incident of Dragendorff”) that the defence, through Augusto António Rocha, sought to take advantage of in court and that motivated a heated exchange of accusations in the press between them.

### Toxicological results

At the time of the case, toxicological analyses always included three phases: i) maceration of the viscera for several hours with alcohol or acidulated water; ii) evaporation of the alcohol; and iii) division of the extracts into several aliquots for subsequent colo­rimetric reactions with “specific” reagents, allowing identifying xenobiotics in the collected crystals. It is important to highlight that there are plant poisons whose colorimetric reactions are confused with those for ptomaines (i.e. cadaveric alkaloids), resulting in irrelevant toxicological results. The seven experts’ reports presented in court regarding the analyses of the viscera and suspicious objects are succinctly detailed below.

#### First report

The first report concerns the toxicological analysis of Mário Guilherme Augusto de Sampaio’s viscera collected in the first autopsy contained in four vials and preserved in alcohol, the urine from the first autopsy stored in another vial, the viscera collected in the second autopsy and a sponge (at those times used to collect existent blood at the thoracic cavity, lungs, and heart), each contained in the respective vial without adding preservative liquid. Experts employed three groups of compounds/reagents for alkaloid analysis:

- Solvents: phenol, petroleum ether, chloroform, and amyl alcohol;
- Precipitants: Bouchardat’s or Wagner’s reagent (i.e. iodine in potassium iodide), Dragendorff’s reagent (i.e. double potassium and bismuth iodide), Valser’s or Mayer’s reagent (solution of potassium mercuric iodide in an excess of potassium iodide), Stein’s reagent (double zinc and potassium iodide), Marmé’s reagent (double cadmium and potassium iodide), Scheibler’s reagent (phosphotungstic acid), Schultze’s reagent (phosphoantimonic acid), Sonnenschein’s or De Vry’s reagent (phosphomolibdic acid), picric acid, tannic acid, mercury chloride, gold chloride, platinum chloride, and ferric chloride; and
- Colouring reagents: sulfuric acid, nitric acid, concentrated and ordinary Fröhde’s reagent, Erdmann’s reagent (nitric-sulfuric acid), Mandelin’s (solution of ammonium vanadate in concentrated sulfuric acid), Lafon’s reagent (selenious acid in concentrated sulfuric acid), Mauricio Robin, Schneider, or Weppen (mixture of sulfuric acid and sugar), Arnold’s reagent (mixture of equal parts of sulfuric acid and phenol), Plugge’s reagent (sulfuric acid followed by potassium nitrite), Otto’s (mixture of sulfuric acid and potassium bichromate), Tattersall (mixture of sulfuric acid and malic acid), Vitali (nitric acid with alcoholic potash), iodine at 15/100, chlorine water, potash bichromate, acetic acid, potash permanganate, very diluted potash nitrogen, ammonia, and sulfuric acid.

The chemical analyses produced forensic conclusions that are faithfully detailed in Table 3. According to a forensic report presented on 7 October 1890, Mário Guilherme Augusto de Sampaio’s death was attributed to morphine and delphinine poisoning [12,13]. Delphinine, like aconitine, is a poison extracted from plants belonging to the Ranunculaceae family and has the ability to act as an allosteric modulator of voltage-gated sodium channels [19,20]. *Delphinium staphisagria* seeds are the main source. It was once used under the name Stavesacre as an herbal treatment for body lice [20], but its cardiotoxicity, in particular, has been described [21].

**Table 3.** Forensic toxicological results of Mário Guilherme Augusto de Sampaio’s biological samples.

Object	Forensic toxicological results
First autopsy	• Narceine and a “small portion” of morphine was registered in urine • The existence of morphine and narceine in the viscera was clearly characterized by chemical analysis • Chemical reactions gave indication of the existence in the same viscera of an organic substance, which based on the chemical characteristics resembled delphinine • The amount of morphine found was very high and could cause the death of a child by itself • Morphine – delays and shortens the pulse, causes a feeling of fullness and distension in the head, drowsiness, or sleep, and sometimes before that, headaches and disturbance of vision. During wakefulness, sensitivity is numb, a gastric malaise is felt, including nausea and even vomiting, difficulty in the emission of urine, constipation, loss of appetite, muscle weakness, exaggerated sweating. When the dose is elevated and toxic, disorders of the nervous system assume an apoplectic form, there is loss of senses, which causes a comatose state, noisy breathing, and sometimes seizures before death • Narceine (i.e. also an opium alkaloid) – a powerful hypnotic drug for man. The sleep produced is deeper and much quieter than that of morphine. Unlike morphine, it seems to make nausea and vomiting disappear, but causes bladder paralysis, irritates urinary organs and produces catarrhal nephritis • Delphinine – effects include local excitation of the mucous membrane of the digestive tract, giving rise to salivation, nausea, vomiting and intestinal pain; the excitation of the brain determines the decrease in the number of cardiac pulsations; excitation of the spinal cord, followed by paralysis; paralysis of the brain, which determines death by asphyxiation; paralysis of the excitatory ganglia of the myocardium, which determines the decrease and weakening of the heartbeat, leaving this organ in diastole at the time of death. The anatomical-related lesions include hyperaemia of the digestive tract and engorgement of the viscera by fluid and black blood
Exhumation	• It confirmed the presence of morphine and narceine and also corroborated the presumptive existence of delphinine • Investigations of mineral toxics, free phosphorus, and hydrocyanic acid (prussic acid) produced negative results

#### Second report

The second report, which was presented few days later, on 20 October 1890, concerned the chemical analysis of the vials seized at Vicente Urbino de Freitas’ home. The experts concluded that the first vial was a hydroalcoholic solution of cocaine hydrochloride; those numbered as 2, 3, and 8 were aqueous solutions of cocaine hydrochloride; the matter in vial number 5 was glycerine and a small portion of cocaine hydrochloride; the product in vial number 6 was a silver nitrogen solution, already partly altered; vial number 4 contained a mercurial ointment, fluidised by sweet almond oil or other similar oil; and finally, vial number 7 contained a mercurial nitrogen solution. Therefore, none of the substances analysed were related to the alkaloids found in Mário Guilherme Augusto de Sampaio’s viscera.

#### Third report

On 10 December 1890, experts presented the third report related to the toxicological analysis of the almonds sent to Berta Fernanda Sampaio. The conclusion was that they did not contain toxic or harmful substances and displayed the normal composition corresponding to good industrial products called by that name.

#### Fourth report

On 20 December 1890, the fourth report on the stamps offered by Vicente Urbino de Freitas to Mário Guilherme Augusto de Sampaio was presented. The conclusion revealed that they were not impregnated with toxic organic or mineral substances.

#### Fifth report

The fifth report was also submitted on 20 December 1890, and concerned the toxicological analysis performed on the syringe used for the enema admini­stration to Mário Guilherme Augusto de Sampaio by his uncle. Experts declared that this object, after use, was thoroughly washed. They concluded that it did not contain toxic mineral or organic substances.

#### Sixth report

The sixth report concerned the analysis of the viscera from the corpse of José António de Sampaio Junior. The results, presented on 31 December 1890, are faithfully detailed in Table 4 [12,13].

**Table 4.** Forensic toxicological results of José António de Sampaio Junior’s biological samples.

No. Toxicological report
Viscera results were negative for mineral poisons and hydrocyanic acid Since the viscera were in an advanced state of putrefaction, the chemical analysis revealed the existence of alkaloid materials of the class of ptomaines, and not of natural plant alkaloids In those circumstances, the results of the toxicological analysis, without denying the hypothesis of poisoning by plant alkaloids since in such cases the search for these compounds may be impossible, do not provide any new argument in favour of this presumption

#### Seventh report

The seventh and final report, presented on 12 January 1891, concerned the toxicological analysis performed on stains found on the mattress on which José António de Sampaio Junior died. In those stains, mine­ral or organic toxic matters were not found in appreciable quantities, and direct examination showed that at least some of the stains were not the result of vomit.

## The contradictory toxicological reports

**Figure 10.**
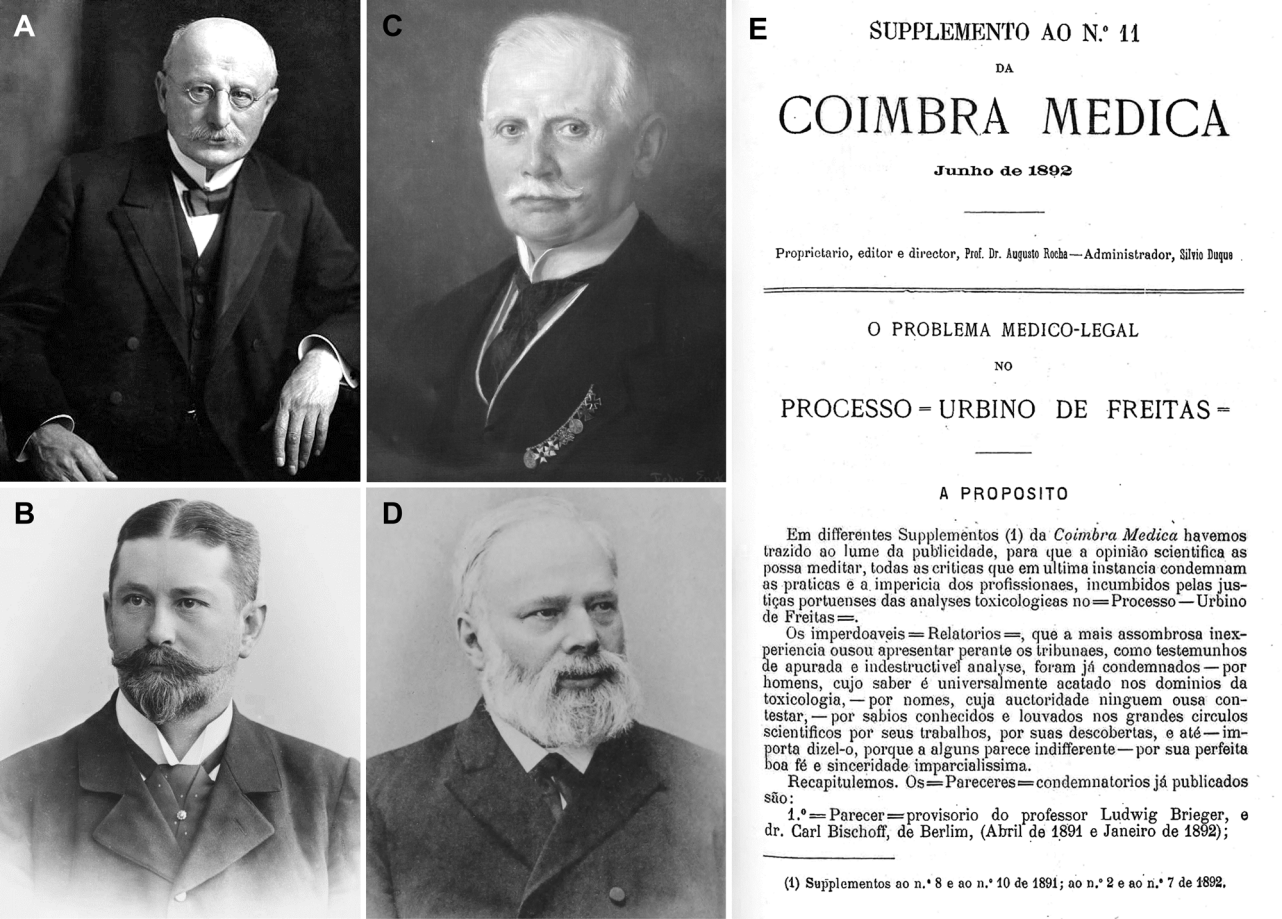
Recovered portraits of (A) Ludwig Brieger, (B) Carl Adam Bischoff, (C) Heinrich Beckurts, and (D) Theodor Gottfried Valentin Husemann. (E) The cover of the supplement to the *Coimbra Médica* n°11.

**Figure 11.**
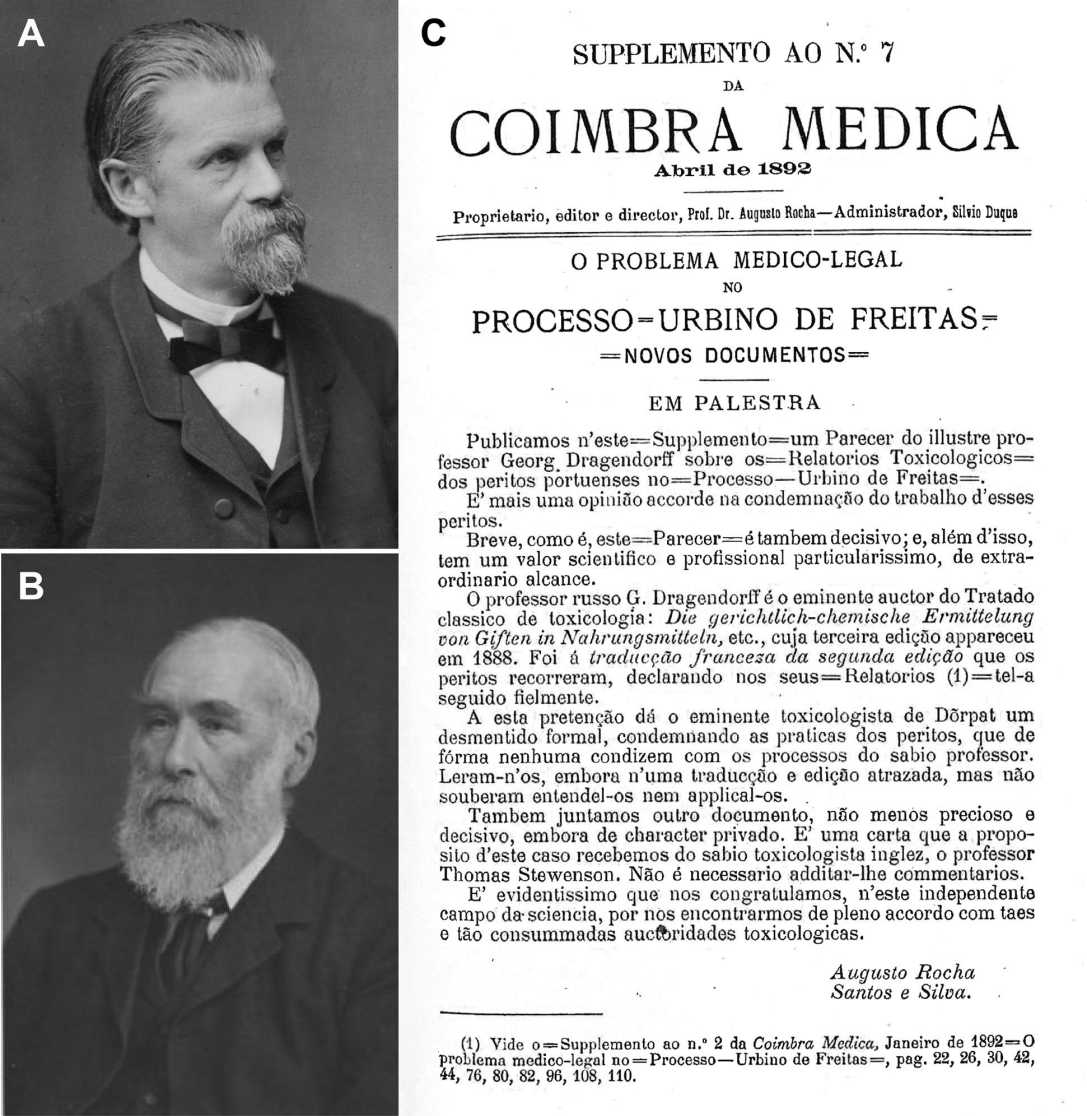
Recovered portraits of (A) Johann Georg Noel Dragendorff and (B) Thomas Stevenson. (C) The cover of the supplement to the *Coimbra Médica* n°7.

**Figure 12.**
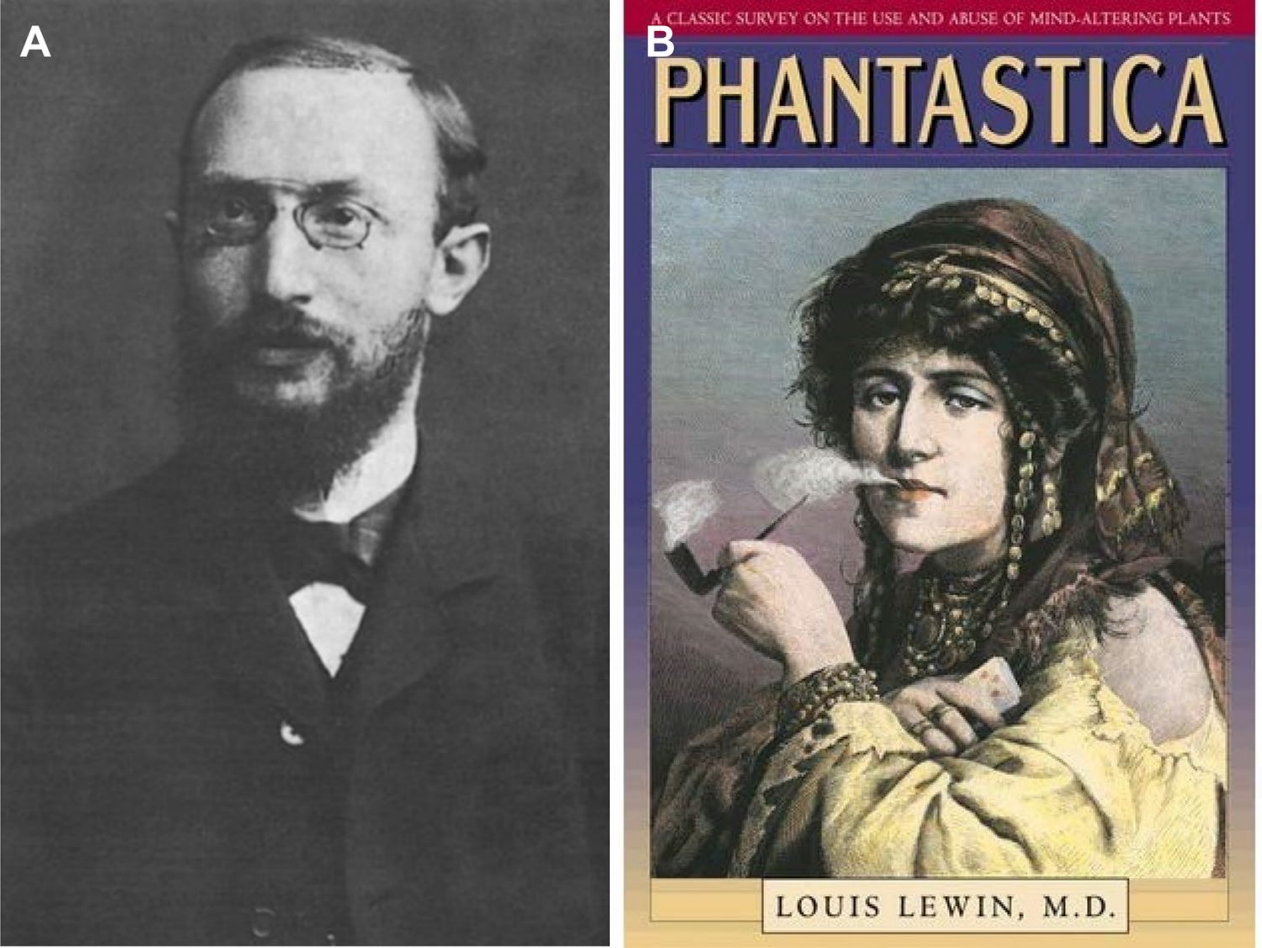
(A) Portrait of Louis Lewin. (B) The cover of his famous book entitled “Phantasia”.

**Figure 13.**
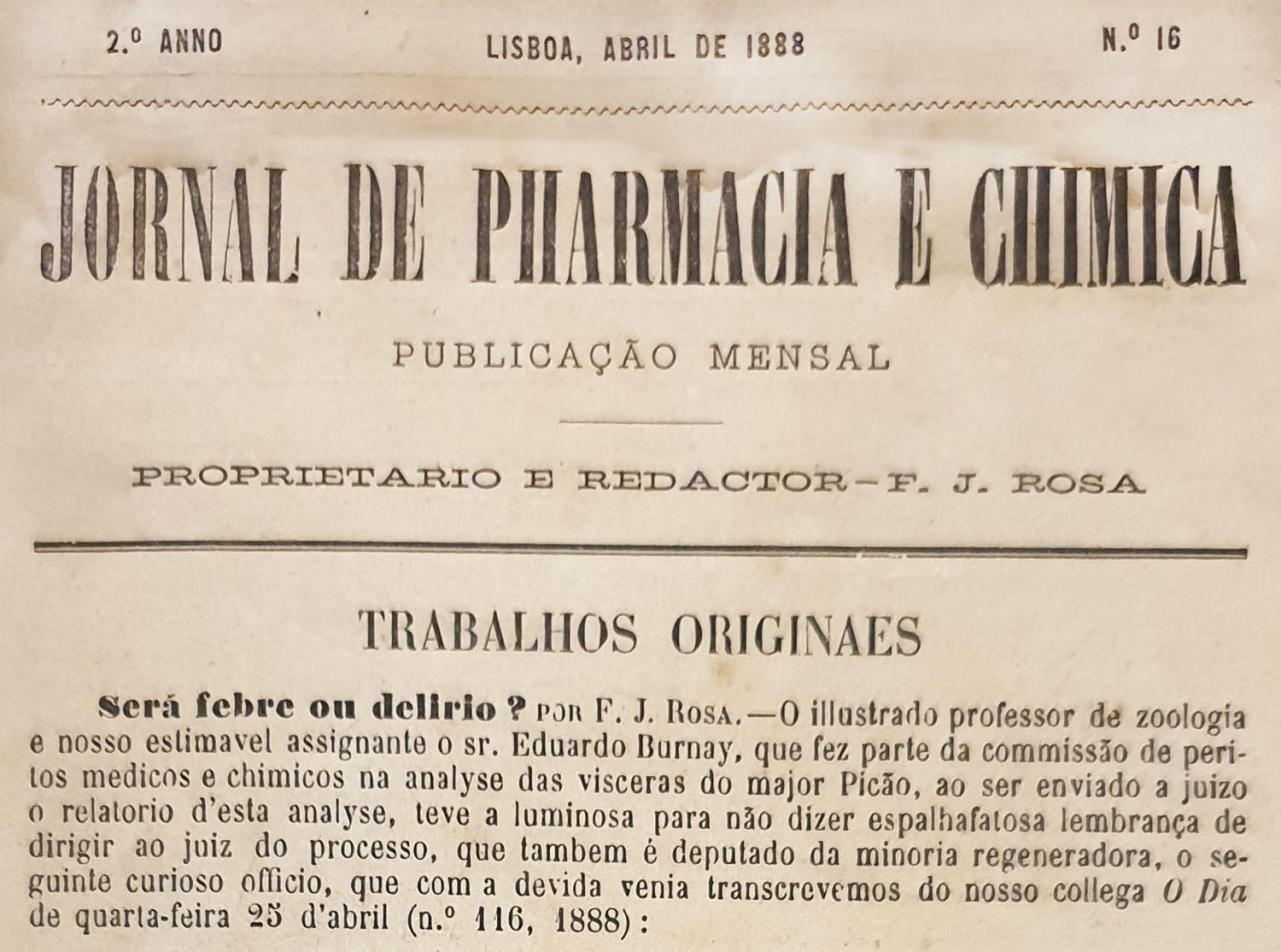
Cover of the *Jornal de Pharmacia e Chimica* edited by Francisco João Rosa.

These contradictory reports gave rise to great scientific controversy, which sometimes led to personal confrontation. Indeed, the controversy increased when Vicente Urbino de Freitas’ defence lawyers, following an appeal to the Porto Court of Appeal, recruited Augusto António Rocha (Figure 9A). He agreed to help on two conditions: i) he would need to work with Joaquim dos Santos e Silva (1842–1906; Figure 9B) and ii) he needed to consult the eminent foreign toxicologists Hugo Mastbaum (Figure 9C), Raimundo da Silva Mota (Figure 9D), Ludwig Brieger (Figure 10A), Carl Adam Bischoff (Figure 10B; [22]), and Heinrich Beckurts (Figure 10C). However, many other prominent European toxicologists became involved in the case, such as Theodor Gottfried Valentin Husemann (Figure 10D), Johann Georg Noel Dragendorff (Figure 11A), Thomas Stevenson (Figure 11B), Louis Lewin (Figure 12; [23]), and Francisco João Rosa (Figure 13 provides the cover of the *Jornal de Pharmacia e Chimica* edited by him). Expert opi­nions of some of these toxicologists were published in several Supplements of *Coimbra Médica* (Figures 9E, 10E, and 11C provide some copies of the covers together with respective experts).

**Figure 14.**
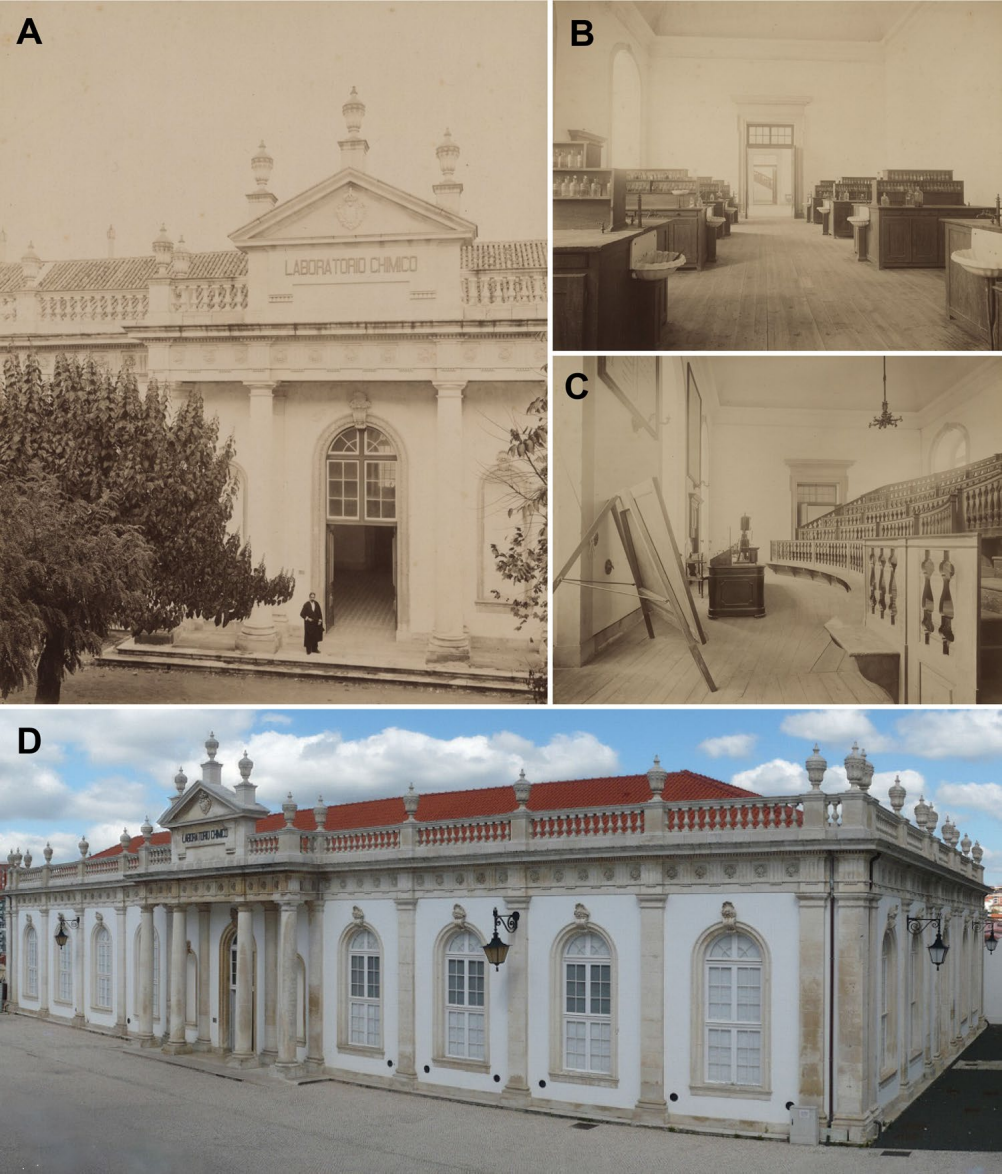
(A) Front view of the Chemistry Laboratory of the University of Coimbra, (B) its chemistry laboratory and (C) its amphitheatre, dating from 1899. (D) The contemporary view is also provided. Photos A, B, and C were obtained by Augusto Bobone in 1892, who was the official photographer for the Royal House in the last years of the Constitutional Monarchy.

**Figure 15.**
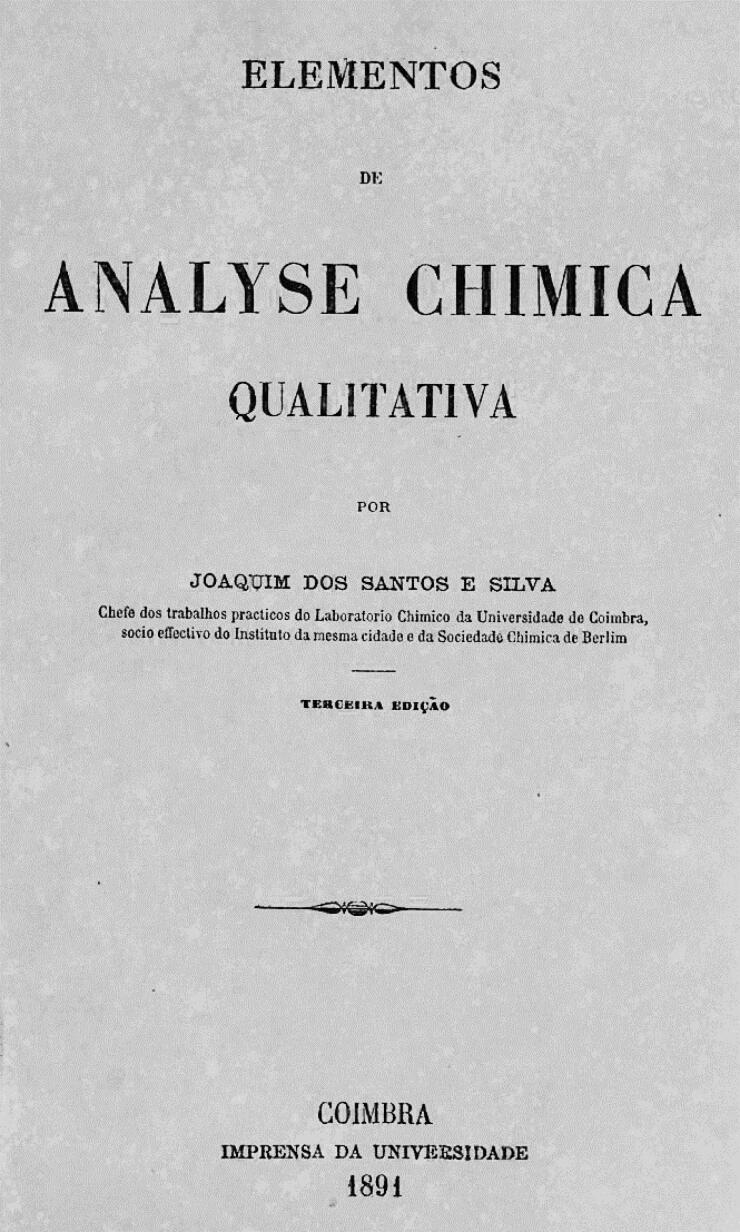
Cover of the book entitled *Elements of Qualitative Chemical Analysis* by Joaquim dos Santos e Silva [24].

The main initiator of this campaign was Augusto António da Rocha, supported by Joaquim dos Santos e Silva, who was a highly respected pharmacist, toxi­cologist, and chemist at the Chemistry Laboratory of the University of Coimbra (Figure 14). These experts issued a report dated 18 April 1891 in the supplement to issue n°8 of the *Coimbra Médica* journal, together with the reports of the wise German experts noted in Figure 10. The report mentioned that the applied chemical analyses correspond to “typical reactions of ptomaines”. Here are their words: “readers…they should not forget for sure that the toxics incriminated by the experts were morphine and delphinine, with narceine, not reputed by themselves as toxic, but rather as a hypnotic of great energy, the most ­powerful of all opium bases, and intended to mask the effects of the poisons, to misdirect the expertise of doctors and chemists, who have the responsibility of shedding light on this monumental case. They were able to convince the audience, who was powerfully thrilled and madly excited from the first acts of the drama. The general opinion applauded the skill, the talent, the science of those teachers….” His friendship with Vicente Urbino de Freitas certainly costed Augusto António da Rocha a significant sum of money. Joaquim dos Santos e Silva claimed that the reactions employed by the Porto experts to characterise morphine had lost importance, as presented in his textbook [24,25] (Figure 15).

There were several other contradictory opinions on the toxicological results obtained by the official experts, whose major points are summarised in Table 5, and several of them were published in the *Coimbra Médica*. All, in one way or another, refer to the conclusions obtained by the official experts whose many inaccuracies and false procedures precluded elucidation of the cause of Mário Guilherme Augusto de Sampaio’s death [8,12,13,26,27]. All these consultations were unfavourable to the work and conclusions of the official experts, extensively highlighting the errors and the presence of putrefaction products that were allegedly confused with toxic plant alkaloids, as well as the abuse and misunderstanding of the use of analytical methods.

**Table 5.** Forensic toxicological reports of contradictory experts on the Vicente Urbino de Freitas forensic case.

Experts	Forensic toxicological reports
Ludwig Brieger (1849–1919) and Carl Adam Bischoff (1855–1908)	• Opinions published in the Supplement of *Coimbra Médica* n°8, April 1891 • Ludwig Brieger – professor of Internal Medicine at the Berlin School of Medicine • Carl Adam Bischoff – first toxicologist for the medical-legal section of the Berlin Police Prefecture • The colourimetric reactions were not caused by the presence of an alkaloid but by the amyl alcohol impurities, which was not freshly distilled or purified; and also, by the abundant septic products (presumed ptomains) of the cadaver due to the limitations of the chosen method for the isolation and purification of extracts • The intended evidence of delphinine is wrong • The presence of morphine is also rebuttable • As for narceine, the reactions that lead to its detection are futile, and nothing more than reactions of ptomaines
Hugo Mastbaum (1859–1945)	• Analytical chemist, assistant of Carl Adam Bischoff at the Faculty of Sciences of Berlin, who at that time resided in Portugal as an analytical chemist and the Director of the Chemistry Laboratory of the 7th Agronomic Region of Lisbon • Changed his focus from chemistry to historical notes and obituaries • One of the founders and first secretary of the Portuguese Society of Chemistry • Opinion published in a Supplement of *Coimbra Médica* n°8, April 1891 dated of November 16, 1891 • He believed that the reports were full of defects and that in chemistry, their conclusions were completely null
Heinrich Beckurts (1855–1929)	• Professor at Brunswick Polytechnic School • Opinion published in a Supplement of *Coimbra Médica* n°8, April 1891 • The colourimetric reactions were produced by the presence of impure substances of an alkaloid nature (the so-called ptomaines of Selmi) • In the viscera, there was neither morphine, narceine, nor delphinine
Theodor Gottfried Valentin Husemann (1833–1901)	• Physician, professor of therapy of the University of Göttingen • Opinion published in a Supplement of *Coimbra Médica* n°11, June 1892 • The alkaloids found are no more than ptomains resulting from putrefaction
Johann Georg Noel Dragendorff (1836–1898)	• German Dorpat professor, pharmacist, and chemist • Developed the popular Dragendorff’s reagent for colorimetric test with alkaloids, producing a reddish orange precipitate • Opinion published in a Supplement of *Coimbra Médica* n°7, April 1892 • He believed that the four Porto experts abusively made use of the analytical methods that he developed and that they did not consider the interference of putrefaction products
Thomas Stevenson (1838–1908)	• Toxicologist, forensic chemist, and expert in English medical jurisprudence • Opinion published in a Supplement of *Coimbra Médica* n°7, April 1892 • He believed the four Porto experts were not authorised to conclude that Mário Guilherme Augusto de Sampaio, the daughter of Vicente Urbino de Freitas, and José António de Sampaio Junior were poisoned by morphine, narceine and delphinine, due to putrefaction
Louis Lewin (1850–1929)	• German pharmacologist, toxicologist, and medical historian • Published the first methodical analysis of Peyote cactus, causing a variant to be named Anhalonium lewinii in his honour • Published the well-known historical book Phantastica (1924), which began an era of ethnobotany that continues to the present day. He is considered the father of the study of psychoactive plants • Opinion in a Supplement of *Coimbra Médica* n°11, June 1892 • He acquired the scientific conviction that the chemical analyses of the cadaveric parts in no way demonstrated that the products obtained from urine and viscera of Mário Guilherme Augusto de Sampaio, the daughter of Vicente Urbino de Freitas, and José António de Sampaio Junior, were vegetable alkaloids
Francisco João Rosa	• Analyst of the Lisbon Hygienic Laboratory, known at the time as the Central Institute of Hygiene (now the Doctor Ricardo Jorge National Health Institute), participated in this process with his colleagues from Coimbra • Editor of the scientific *Jornal de Pharmacia e Chimica* • Opinion published in a Supplement to *Coimbra Médica* n°11, June 1892 • The toxicological reports have serious defects and inexcusable errors • Corroborated the opinions of foreign experts and questioned the integrity of the chain of custody and the integrity of the analysed samples • Nevertheless, he firstly declared in 1891 that he was not enough competent to evaluate the mistakes of Porto experts. He also said that the work “was herculean and remarkable as no one else ever done in Portugal or likely abroad”, condemned the “doctor Vicente Urbino de Freitas as an exceptional criminal” calling him “a new Jack specie”
Raimundo da Silva Mota (1840–1910)	• Professor of Pathological Anatomy, Faculty of Medicine, University of Coimbra • First defence witness called to offer insights into the conclusions of the toxicological tests

## It was not an easy life for the toxicologist António Joaquim Ferreira da Silva

Toxicological work related to the Vicente Urbino de Freitas case practically chained António Joaquim Ferreira da Silva to the laboratory between 1890 and 1893, leading him to successive analyses and several discoveries, some of which are highlighted herein:

- Characterisation of the reaction to detect cocaine, since at the time the identification of the drug was primarily based on observing its physiological effects [28]. This reaction, presented by Pierre Eugène Marcellin Berthelot in the 18 August 1890 session of the Paris Academy of Sciences, consisted of evaporating to dryness a solution containing cocaine in the presence of smoking nitric acid and then heating the residue with potassium hydro­xide alcohol solution, forming a volatile substance with a smell resembling peppermint. Subsequently, this substance was identified as ethyl benzoate. This reaction made it possible to distinguish cocaine from many other alkaloids, such as atropine, brucine, cinchonine, codeine, delphinine, eserine, hyoscyamine, sabadiline, strychnine, and veratrine, all very toxic alkaloids. António Joaquim Ferreira da Silva established the sensitivity of the reaction, having verified that he could identify down to a “half-decimilligram” (i.e. 50 μg) of cocaine hydrochloride;
- Characterization of the reactions that extended the use of ammonium selenium to detect other alkaloids [29], increasing the application of the reagent proposed by Philippe Lafon. This work was presented by Charles Friedel in the 1 June 1890 session of the Paris Academy of Sciences. In 1885, Philippe Lafon identified a new reagent for morphine and codeine: ammonium selenite in sulfuric acid [30]. The reagent was prepared by dissolving 1 g ammonium selenium [proba­bly (NH_4_)_2_SeO_3_·H_2_O], in 20 g concentrated sulfuric acid (selenious acid in concentrated sulfuric acid). This reagent turned a green colour in the presence of those alkaloids but not with the other alkaloids obtained from opium, nor with ptomaines formed in the putrefaction of cadavers. António Joaquim Ferreira da Silva modified the composition of the Lafon reagent (or Mecke’s reagent) starting from 1 g ammonium selenium to 25 g or 50 g. What was a reagent of only two alkaloids became a reagent of alkaloids in general, such as berberine, escrine (physostigmine), narcotine (noscapine), papaverine, sobanin, and narceine. The Ferreira da Silva’s reagent, as it has been called since 1896 by E. Merck (chemical company; “E” is Emanuel’s initial, who was a pharmacist), or the Lafon and Ferreira da Silva’s reagent as António Joaquim Ferreira da Silva called it, turns morphine, for example, into blue-green or green;
- Discovery of new reactions to detect eserine [31]. When a small eserine crystal in a porcelain capsule is dissolved in a drop of smoking nitric acid, a pale-yellow solution is obtained which, when heated in a water bath, successively changes to a more intense yellow and then orange before evaporating to dryness. Constant mixing with a glass rod for one to two minutes until the liquid is eliminated after the exsiccation yields a change in the colour of the residue, which goes from yellow to green. According to António Joaquim Ferreira da Silva, this reaction, which is characteristic of eserine, has a limit of detection of 5 mg.

These congress communications/presentations show that António Joaquim Ferreira da Silva did not act lightly but worked with the forthrightness of a good toxicology expert. He developed experiments and immediately sought the opinion of his peers, putting to discussion his proposals and seeking the legitimacy of the international scientific community. Especially for António Joaquim Ferreira da Silva, who was responsible for the toxicological examination, it was unpleasant to be attacked so many times and see his scientific reputation questioned. Despite this, the result was favourable to him both from the court and peer chemists, who legitimised the scientific suitability of his works presented at the Paris Academy of Sciences. The scientific controversy would not end there, as several researchers continued looking for new methods to overcome possible ptomaine contamination in morphine detection and questioned the results obtained in the case of Vicente Urbino de Freitas [32].

An outstanding advance in toxicology occurred in the context of the medical-legal case of Vicente Urbino de Freitas. The forensic study became a refe­rence in the field because of the long, thorough, and persevering research conducted to identify morphine, narceine, and delphinine. It also led António Joaquim Ferreira da Silva to discover new reactions for serine, cocaine, and other alkaloids. This process would become a fundamental element of information and credibility to the national toxicology field, exposing the technical and scientific capacity of António Joaquim Ferreira da Silva to innovate. The diligence shown by António Joaquim Ferreira da Silva in implementing new methods and equipment and by confronting theoretical and experimental positions inaugurated a new phase of toxicology and forensic chemistry in Portugal. The intervention in the Vicente Urbino de Freitas case, with the results of the analyses being decisive in the trial outcome, added prestige to the laboratory and to António Joaquim Ferreira da Silva. António Joaquim Ferreira da Silva then received successive requests from the courts for new medical-legal cases, which led the Porto City Hall to approve funds for novel toxicological equipments and physiological experimentation.

## Psychiatric report

**Figure 16.**
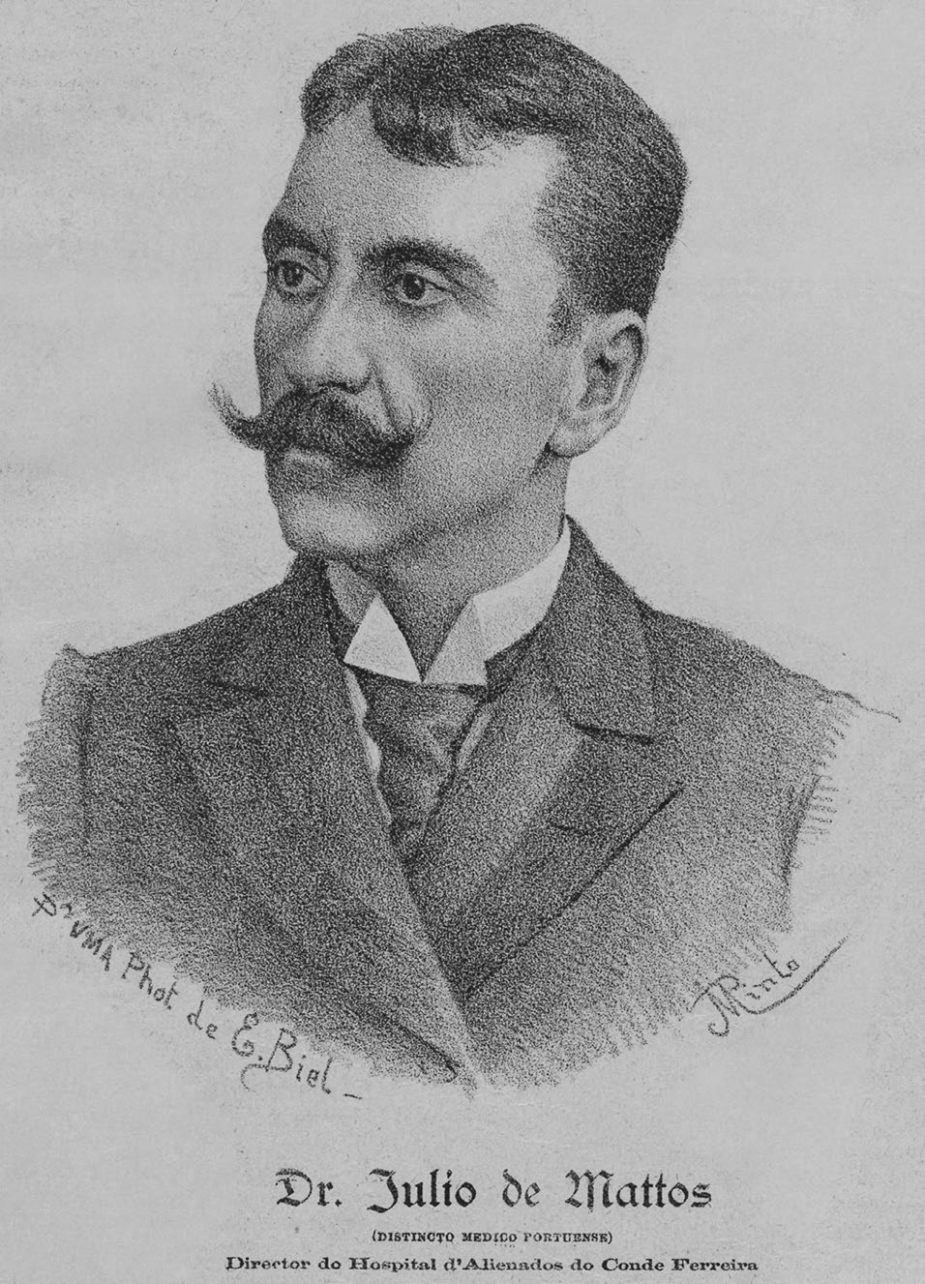
Recovered portrait of Júlio Xavier de Matos.

Júlio Xavier de Matos, a former student of Vicente Urbino de Freitas, participated in the psychiatric analysis (Figure 16). However, his report was not considered in court, most likely because it was unknown or not scientifically recognised at the date of the facts. Júlio Xavier de Matos graduated with a degree in medicine from the Medical-Surgical School of Porto in 1880 with an inaugural dissertation entitled The Pathogenesis of Hallucinations. He was the first Professor of Psychiatry at the Faculty of Medicine of Porto (a position occupied only for a few months in 1911) and Director of the Conde Ferreira Hospital in the same city between 1892 and 1911. Then, he moved to Lisbon to be director of the Rilhafoles Hospital (later called Miguel Bombarda Hospital), and by inherency he assumed the responsibility of the Psychiatry Curricular Unit of the Faculty of Medicine of Lisbon following the tragic death of Miguel Bombarda in 1910. Between 1899 and 1911, he held the position of alienist physician of the Medical-Legal Council of Porto and later of Lisbon. Júlio Xavier de Matos was the first major Portuguese psychiatrist and a pioneer of clinical and medical-forensic studies of Psychiatry in Portugal.

**Figure 17.**
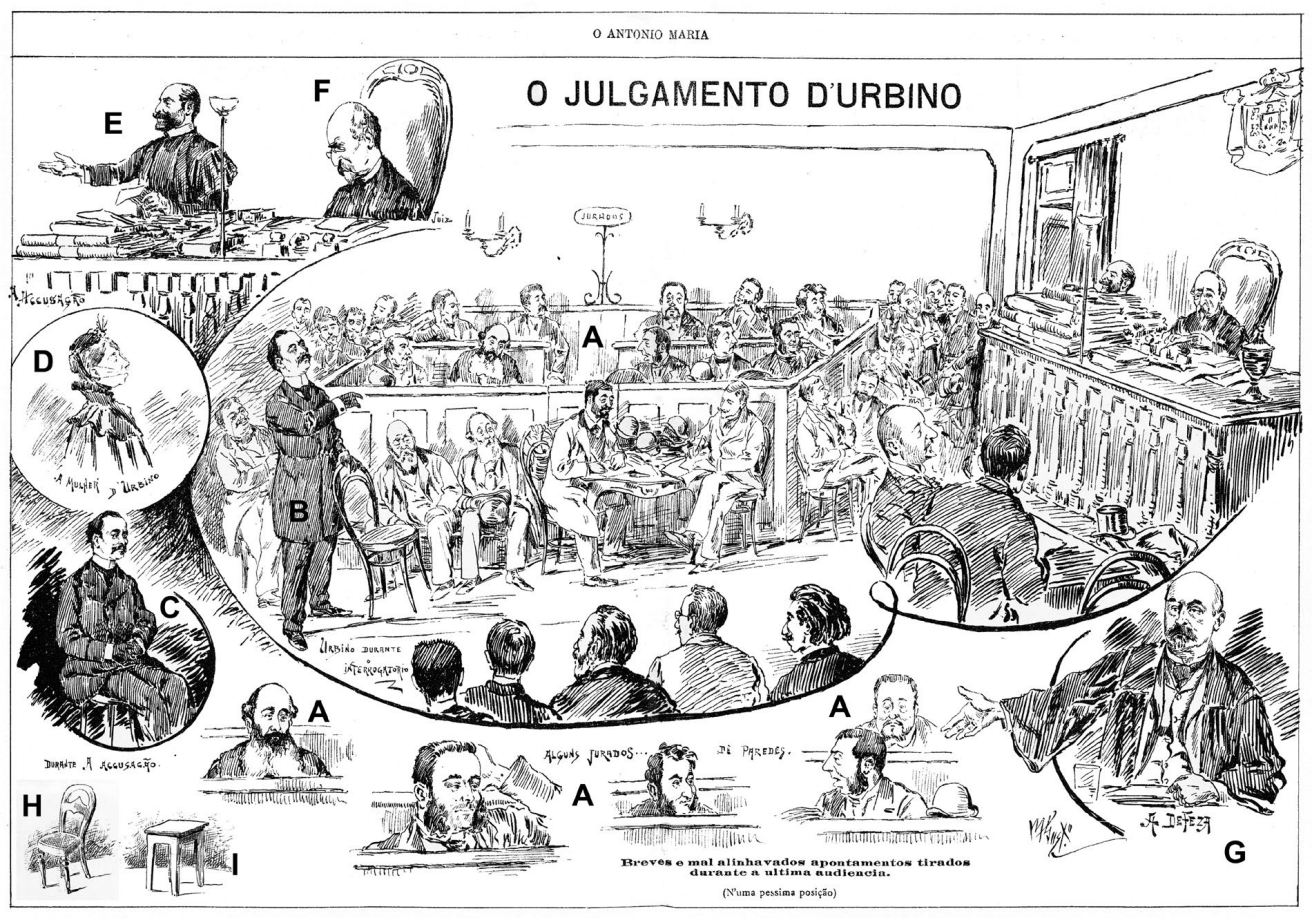
Graphical report of the Vicente Urbino de Freitas trial. (A) Around the central image in an approximately elliptical format, enlarged portraits of some individuals sitting on the bench are distributed, indicated as “jurors”. (B) The defendant is depicted in the central image standing on the left side with an overcoat and black gloves. (C) The defendant is also illustrated sitting in a round picture on the left. Above this picture, a portrait of Vicente Urbino de Freitas’s wife is presented (D), and at the top of this sequence, the prosecutor (E) and the judge (F) are shown. (G) The Vicente Urbino de Freitas defence lawyer is in the lower right corner. The seat of the rich (H) and the poor (I) people is an interesting distinction.

In excerpts that refer to the entry of Vicente Urbino de Freitas in the court at the beginning of the hearings, the character’s physiognomy was described as a consequence of the perverse nature of the crimes: “the defendant passed through the crowd, pale, head up, almost without looking at anyone” [11]. At the hearing on 23 November 1890, the accused appeared at 9:45 a.m., impassive in appearance and entering the room with sombre glances. The mentioned characteristics also echoed the “patibular” portrait that attributes the physiognomy of the criminal man to a gloomy and gruesome appearance [33]. The portrayal of the character of Vicente Urbino de Freitas received important contributions from the 18th century theoretical and scientific discourse that described him as a cold, calculating, intelligent, manipulative, and professionally prestigious figure. Nevertheless, it is impor­tant to remember that the roots of this repertoire drank in an imagery produced by both scientific and fictional texts, since they all aimed to recreate a criminal stereotype [34]. Curiously, Vicent Urbino de Freitas was treated with respect in court. Indeed, in a graphic report entitled “o julgamento d’Urbino” (translation to Urbino’s trial) [35], the illustrator’s presenting of the first moments of the audience, sat Vicente Urbino de Freitas in the chair of the rich defendants (Figure 17).

For Júlio Xavier de Matos, the crime was significantly a pathology that would focus tenaciously on a diffuse (but constitutional) anthropological nature. “Physiognomic expression” was referred by Júlio Xavier de Matos, who analysed the unexpected reactions of the moral crazy/corrupt individuals. According to this psychiatrist, the profile of the genius man fits the symptomatology of moral dementia [36]. In fact, he supported this thesis with references to famous homicide cases with perpetrators that made an instinctive defensive impression on those who knew them [37]. For this reason, a detailed physical description of Vicente Urbino de Freitas is found in his work: “the physiognomic expression has in the moral crazy man or in the non-innate criminal something unsympathetic and repellent, which describes itself with difficulty, but feels for a sort of instinct”. Indeed, for the psychiatrist, “the poisoner Vicente Urbino de Freitas only inspired in students a confused feeling of repulsion. The look, sometimes truculent and vitreous, but other times restless and suspicious; the absence of the eurythmy of the facial lines; the substitution of frank laughter with a cruel or cynical rite, all this contributes to provoking emotions of dislike and sometimes fear”. According to the same physician, the moral crazy man had, from the psychic point of view, a cultivated intelligence generally linked to the sciences and knew how to conduct himself appropriately in society, making use of relational codes [36].

## After conviction: life in Brazil and the return to Portugal

**Figure 18.**
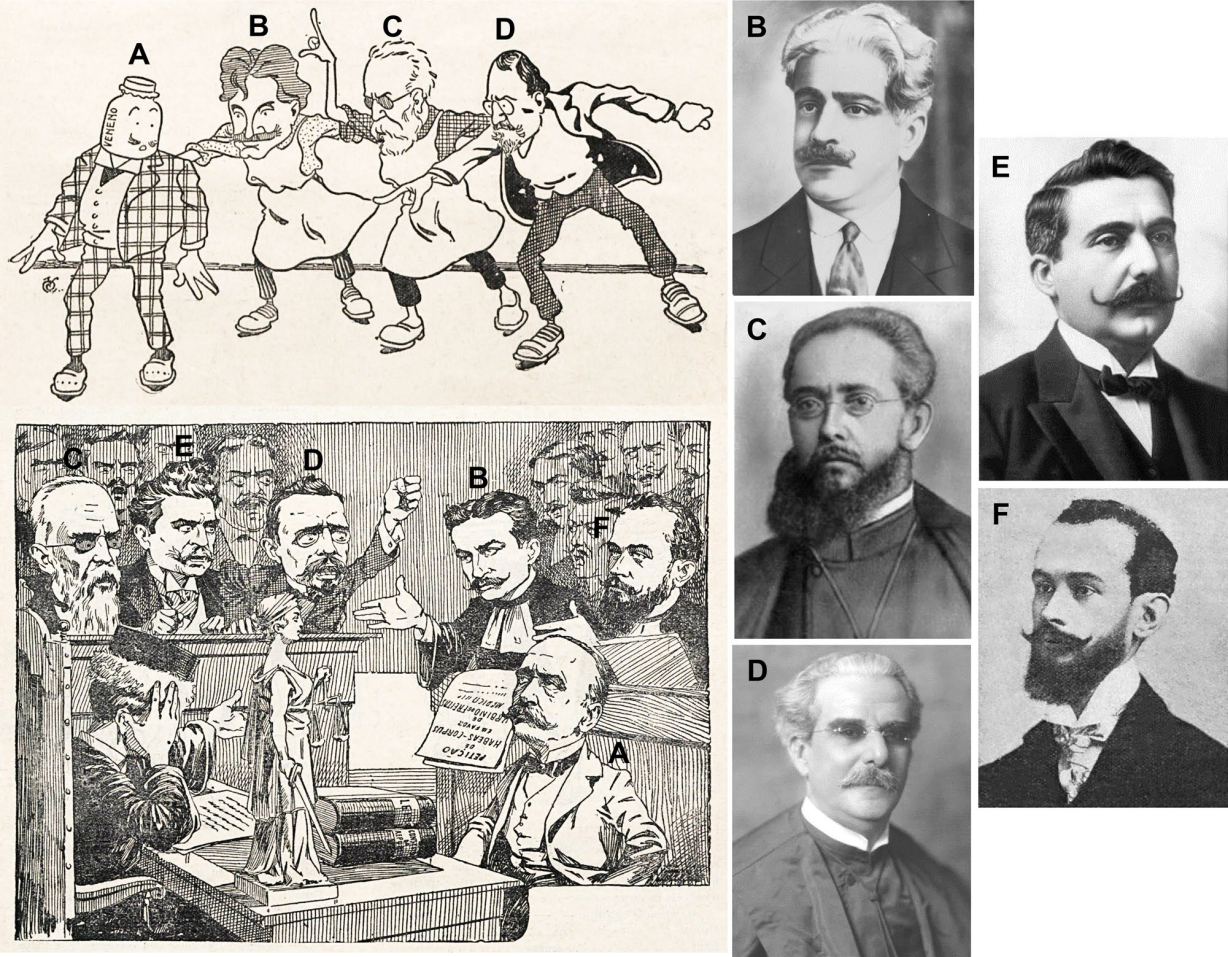
(A) Caricature of Vicente Urbino de Freitas with a bottle of poison on his head. Recovered portraits of (B) Oswaldo Gonçalves Cruz, (C) Alfredo Pinto Vieira de Melo, (D) Cândido Barata Ribeiro, (E) Augusto Tavares de Lyra, and (F) António Moitinho Doria.

**Figure 19.**
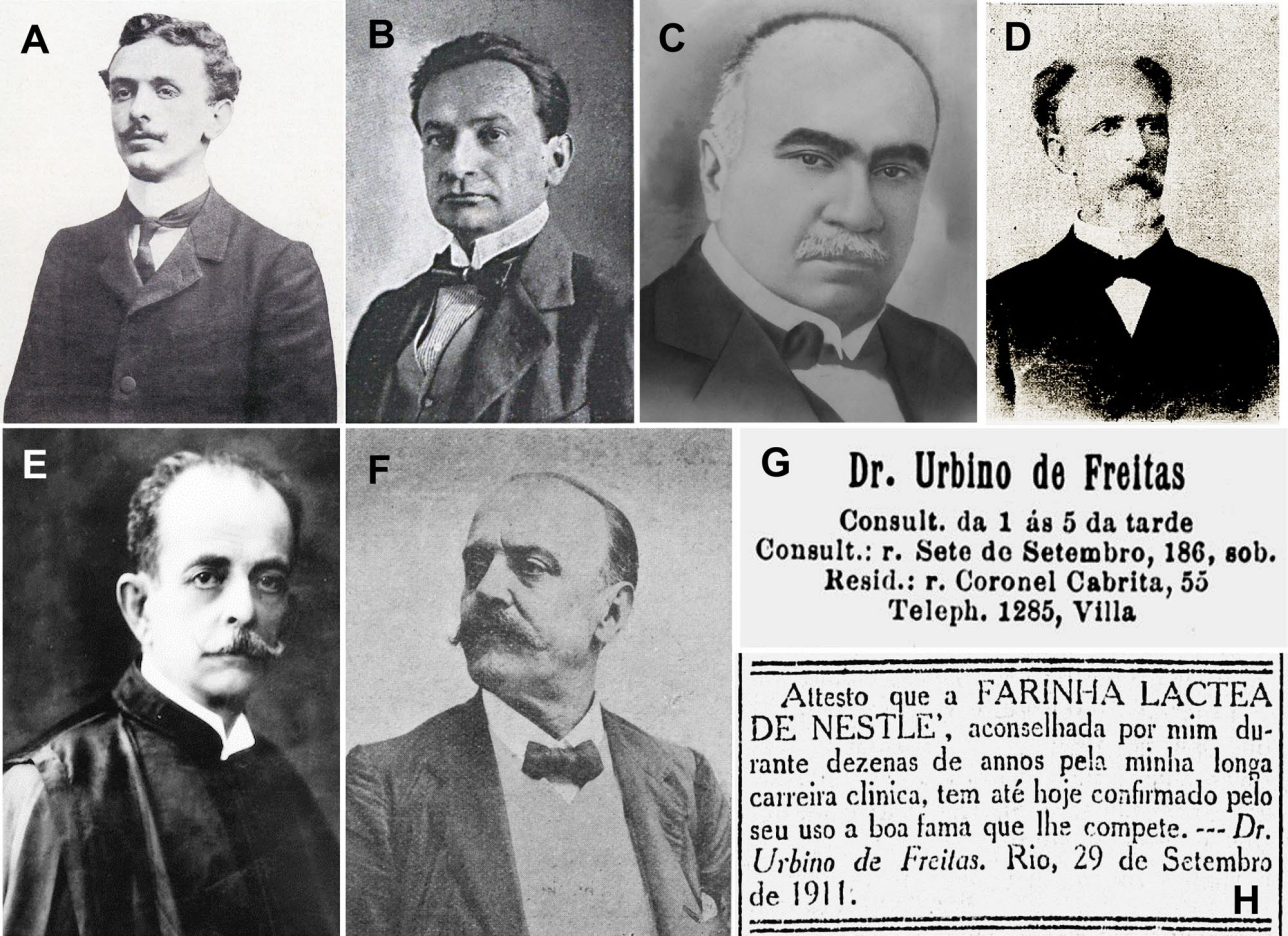
Recovered portraits of (A) Fernando Mendes de Almeida Junior, (B) Celso Bayma, (C) Jaime Pombo Bricio Filho, (D) Alberto Estanisláo, and (E) António Joaquim Pires de Carvalho e Albuquerque. (F) Recovered portrait of Vicente Urbino de Freitas, (G) his clinic and residence addresses in Brazil, and (H) his Nestlé® advertisement.

In Brazil, Vicente Urbino de Freitas (Figure 18A) sought permission to practice medicine twice, first in Campinas and then in Rio de Janeiro; both applications were rejected. In 1906 in Rio de Janeiro, there was a government clamp down on illegal medi­cal practices. Thus, the Director of Public Health, Oswaldo Gonçalves Cruz (1872–1917; Figure 18B), ordered the prosecution of Vicente Urbino de Freitas for illegally practicing medicine and wrote to the local pharmacies prohibiting them from filling his prescriptions. Since Vicente Urbino de Freitas failed to comply with these orders, his expulsion from Brazil was requested. Several other illustrious individuals were involved, including Alfredo Pinto Vieira de Melo (1863–1923; Figure 18C), jurist and Chief of Police of Rio de Janeiro for the government of Afonso Pena, Cândido Barata Ribeiro (1843–1910; Figure 18D), Brazilian physician, politician, and writer, Augusto Tavares de Lyra (1872–1958; Figure 18E), historian, writer, and politician who gave his name to the Tavares de Lyra Institute of Brazil, António Moitinho Doria (1874–1950; Figure 18F), member of the Brazilian Lawyer’s Institute and one of the founders of the Brazilian Lawyer’s Association, Fernando Mendes de Almeida Junior (1882–death’s date unknown; Figure 19A), lawyer and journalist for the *Jornal do Brasil*, Celso Bayma (1874–1935; Figure 19B), state deputy, senator, and professor of universal ­history, Jaime Pombo Bricio Filho (1865–1951; Figure 19C), physician, professor, deputy, and writer that founded and collaborated in several newspapers such as *O Globo*, *Correio da Manhã*, and *Jornal do Brasil*, and Alberto Estanisláo (Figure 19D), commander and editor of *Jornal do Brasil*. Some of them were against (Figure 18) and others were supportive (Figure 19) of the practice of medicine by Vicente Urbino de Freitas. Nevertheless, 5 days before leaving Brazil, Vicente Urbino de Freitas was arrested because the Supreme Court annulled the *habeas corpus* granted by the judge António Joaquim Pires de Carvalho e Albuquerque (1865–1954; Figure 19E) of the fede­ral court, because the magistrate was judged to be incompetent to rule on the unconstitutionality of the expulsion law. After being arrested, he was granted 5 days to leave Brazilian territory. As noted, even in Brazil, Vicente Urbino de Freitas (Figure 19F) had no luck with the law. One day they were in favour of him and the next ordered his expulsion. Curiously, he was the first victim of the expulsion law voted on in the Brazilian congress, a law that was “sleeping” for a very long time on the desks of the Brazilian Congress. Nevertheless, his fame made it possible to open clinics, acquire residences, and be called to advertise various products with therapeutic connotations (Figure 19G).

**Figure 20.**
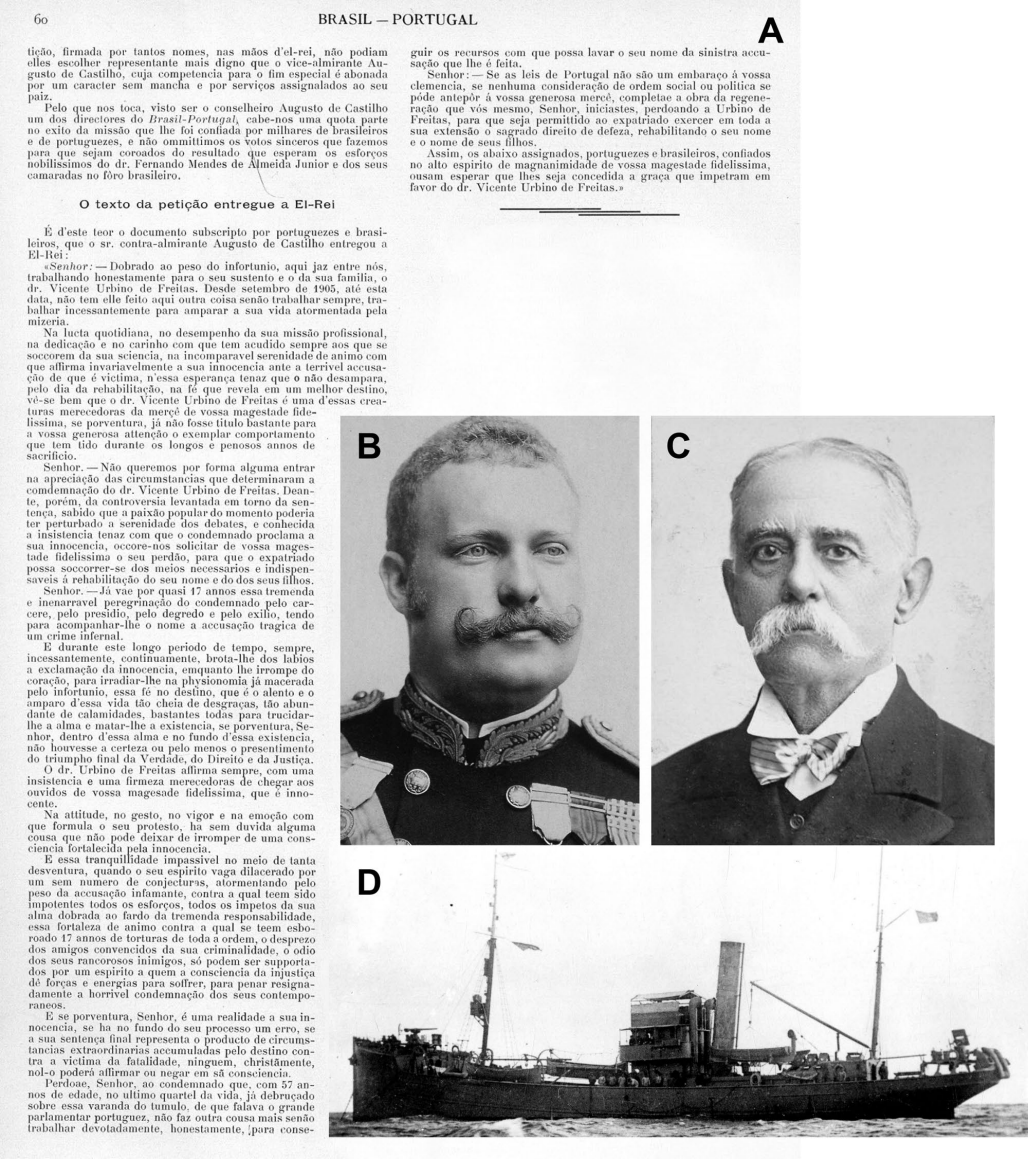
(A) Petition sent to King Carlos requesting a review of the Vicente Urbino de Freitas forensic case. (B) Portrait of King Carlos. (C) The Admiral Augusto Vidal de Castilho Barreto e Noronha delivered the petition, and his name was given to (D) the warship NRP Augusto de Castilho in service of the Portuguese Navy during World War I.

A group of Portuguese and Brazilian citizens, convinced of Vicente Urbino de Freitas innocence, then sent a petition (Figure 20A) to King Carlos (Figure 20B) requesting a review of the case. However, this was not granted. This petition was delivered by Admiral Augusto Vidal de Castilho Barreto e Noronha (1841–1912; Figure 20C), whose name was given to the warship NRP Augusto de Castilho (Figure 20D) in service of the Portuguese Navy during the World War I. It was sunk in combat while escorting the steamer São Miguel, originating the last Portuguese victims of that worldwide military conflict.

## Author’s personal interpretation, concluding remarks, and the beginning of the end

For the final interpretation, it was fundamental to study the life and scientific work of António Joaquim Ferreira da Silva. It is possible to realise the forthrightness of a respectful chemist while simultaneously verifying the dishonesty of Augusto António Rocha. Indeed, the experts of Porto did a remarkable job that was nearly impossible to rebut considering the knowledge of that time. Today, it would be easy to perform irrefutable toxicological analysis, but in those times the steps taken were remarkable. That is why it is particularly difficult to form an opinion on who had performed the best in this titanic fight, while the author is enjoying the forensic knowledge built and grounded on this and other cases for more than 130 years.

**Figure 21.**
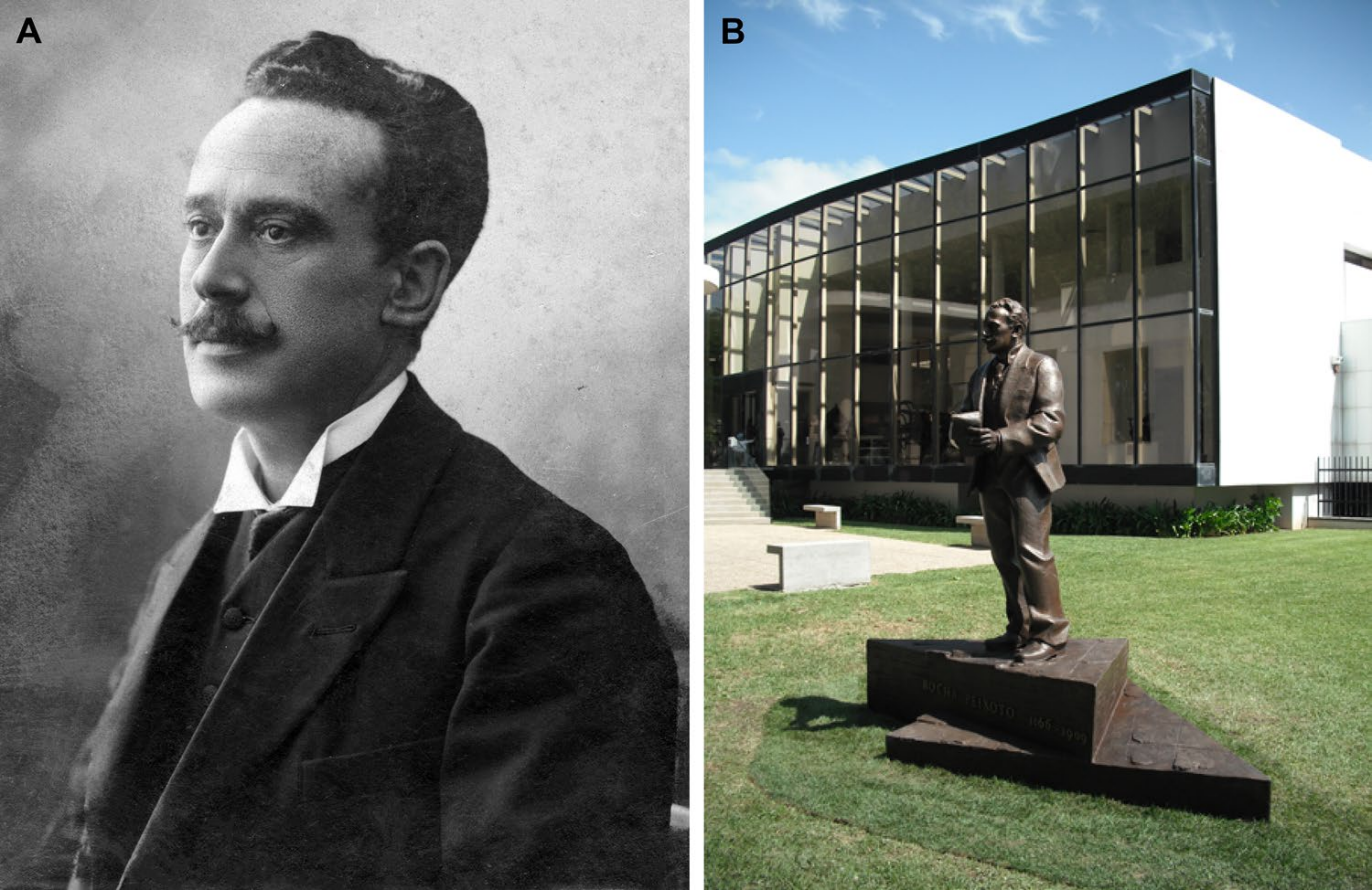
(A) Recovered portrait of Augusto António da Rocha Peixoto and (B) the Municipal Library of Póvoa de Varzim with his name.

António Joaquim Ferreira da Silva was also largely defended by an illustrious naturalist, professor, ethnologist, anthropologist, writer, and journalist, Augusto António da Rocha Peixoto (1866–1909; Figure 21) in his newspaper [38]. Reports, comments, replies, and additional documents that recompose the history and justify the attitude and role of the experts of Porto had just been gathered in a book [12,13,18]. In this regard, it was written that “António Joaquim Ferreira da Silva lives for chemistry, will die for chemistry, and was perhaps born for chemistry; and in this graceful characteri­stic of the already renowned scientific individuality, it is really this trait that marks and distinguishes him: someone characterised by eagerness, sacrifice and heroic persistence. António Joaquim Ferreira da Silva was a good man marked by an outstanding aptitude and undisputed proficiency”.

The report related the toxicological analysis of samples from Mário Guilherme Augusto de Sampaio, opened with a description of the vials and proceeded with the enumeration of the reagents used in the investigation of alkaloids. This preliminary section was followed by the analyses of urine, viscera from the first and second autopsies, the counterproof, the investigation of toxic mineral substances, and the physiological actions of alkaloid products extracted from the urine and viscera of the child. António Joaquim Ferreira da Silva then concluded that the death should be attributed to morphine and delphinine poisoning. The analyses of the liquids seized from Vicente Urbino de Freitas, the almonds sent from Lisbon, the stamps offered by Vicente Urbino de Freitas to his nephew, and the syringe with which he administered the enemas did not reveal toxic mineral or organic substances. The toxicological analysis of the viscera of José António de Sampaio Junior, without refuting the hypothesis of poisoning by plant alkaloids from the advanced state of putrefaction, was postulated to be negative. Together with the toxicological analysis of the mattress where José António de Sampaio Junior died, seven exemplarily reports were prepared by Porto experts and then scrutinized regarding methods, techniques, and chains of custody. Notwithstanding, the campaign to discredit the Porto experts promoted by some Portuguese and foreign toxicologists was strong and aroused intense emotions in the public.

The last babbling words of José António de Sampaio Junior revealed his suspicion that harm was perpetrated against him. Even while treating poor Mário Guilherme Augusto de Sampaio, Vicente Urbino de Freitas himself clearly expressed his scientific conviction that both he and the other children had been poisoned, not accidentally, but by an “infamous attack”. Moreover, none of the doctors called to testify ever hesitated to proclaim the same conviction, even in the immediate pre­sence of the police commissioner. Finally, a confe­rence of thirteen doctors expressed the identical opinion without the slightest discrepancy. In other words, it is improbable that a mistake was made by different medical professionals in a matter of such intense gravity. Curiously, the answer was given by Vicente Urbino de Freitas himself at the trial hearing, stating once again his scientific belief that a poisoning occurred.

The author interviewed a highly renowned Portuguese Judge Counsellor of the Supreme Court of Justice José Pereira da Graça, and he was surprised because the public prosecutor had made the accusation before the report of the thanatological examination was known. However, to date, the integrity of the content of the thanatological examination has never been called into question. In terms of evidence, all the vast circumstantially produced evidence, together with the toxicological expert reports, allowed the court to produce a damning judgment. José Pereira da Graça said that “if it were not for the content of the expert reports, I have no doubt there would not have been a condemnation. This seems so clear to me that the delegate of the Royal Prosecutor did not even accuse Vicente Urbino de Freitas of the homicide of José António de Sampaio Junior at the Hotel Paris, due to inconclusive and even an absence of forensic toxicological results. If, scientifically, it is concluded that the forensic reports are wrong, it does not prove that the crime was not committed, but only that it had not been produced evidence of its committing. Moreover, it is obvious to me that if the court had not provided such probative evidence, he probably would not have been convicted or even would not had been accused or pronounced, assuming that the procedural rules were similar to those which then were in force for many years”. This is an excellent perspective with which I fully identify. After reconstructing the events, recovering amazing historical documents, and understanding the entire story, my doubts were raised by the autopsy reports, especially the toxicological ana­lysis. With current knowledge, it is possible to add further insights if at least one supposed victim could be found. In the beginning, that was just a dream in my mind. Therefore, after 14 years spent searching for the corpse of José António de Sampaio Junior, based on the historical records, which were crossed with the cemetery data, it was finally supposedly found on 27 October 2020. Permission for a new autopsy was obtained and performed more than 130 years after the first major autopsy was executed in Portugal. The cadaver was well preserved. Identification by DNA analysis and comparison with relatives, as well as toxi­cological analyses focusing on narceine, delphinine, morphine, and pilocarpine, will now be performed. The latter compound was mentioned in a letter in *Coimbra Médica*, 1 June 1890 by Vicente Urbino de Freitas as the therapy used for José António de Sampaio Junior. Nevertheless, for him, the death of his brother-in-law was due to the “excesses of various species”. *Melissa officinalis* (i.e. lemon balm)-related compounds will also be analysed.
